# From Passive Matrices to Bioresponsive Interfaces: Polymer-Based Biomaterials for Oral Soft Tissue Augmentation

**DOI:** 10.3390/polym18212746

**Published:** 2026-11-04

**Authors:** Bartłomiej Górski, Natalia Muczkowska

**Affiliations:** 1 Department of Periodontology and Oral Diseases, Medical University of Warsaw, 02-097 Warsaw, Poland; bartlomiej.gorski@wum.edu.pl; 2 Department of Oral Surgery, Medical University of Warsaw, 02-097 Warsaw, Poland

**Keywords:** soft tissue augmentation, connective tissue graft, collagen matrix, keratinized mucosa, transmucosal seal, responsive polymers, platelet-rich fibrin, peri-implant soft tissue

## Abstract

Soft tissue deficiencies around teeth and dental implants remain a common clinical challenge affecting aesthetics, tissue stability, and long-term maintenance. Polymer-based substitutes reduce donor-site morbidity compared with autogenous connective tissue grafts, but comparative evidence frequently shows lower gains in mucosal thickness and keratinized tissue dimensions. This narrative review examines the biological and material factors that may limit the performance of current soft tissue substitutes and evaluates strategies aimed at overcoming these limitations. Particular attention is given to the mismatch between healing dynamics and passive material behavior, including early burst release of bioactive agents, degradation uncoupled from cellular colonization, delivery kinetics that are not designed to respond to the state of the wound, and limited dose control. Current approaches such as platelet concentrates, recombinant growth factors, affinity-based delivery, covalent immobilization, and cell-seeded matrices can improve selected healing outcomes, but their effects remain strongly dependent on the carrier and have not consistently matched the gains in mucosal thickness and keratinized tissue width obtained with autogenous grafts. Stimuli-responsive and bioresponsive polymer systems offer a potential strategy for making degradation or release depend on the local wound state, although their application in oral soft tissue augmentation remains predominantly preclinical. Future research should focus on polymer–cell and polymer–bioactive agent systems in which degradation, cellular colonization, signal delivery, and mechanical performance are better aligned with tissue healing and long-term maintenance.

## 1. Introduction

### 1.1. Soft Tissue Deficiency as a Common Challenge Across Different Clinical Settings

Oral soft tissue augmentation is performed in three distinct situations that are described separately, although they share a common biological and material challenge: the need to increase soft tissue dimensions and maintain the achieved gain over time. Gingival recession around natural teeth exposes the root surface and may result in hypersensitivity, aesthetic complaints, root caries, and increased susceptibility to further attachment loss. A thin periodontal phenotype, characterized by reduced tissue thickness and often a limited width of keratinized tissue, may increase susceptibility to recession and influence the response to restorative, orthodontic, and surgical interventions. Peri-implant soft tissue deficiency presents a related challenge, where the augmented tissue must not only form but also establish and maintain a stable biological barrier.

These clinical scenarios are linked by phenotype and by a common therapeutic objective. Root coverage, phenotype modification, and peri-implant soft tissue augmentation differ in their clinical indication and biological environment, but all aim to increase tissue thickness, keratinized tissue dimensions, or soft tissue volume and to preserve these changes over time [1,2]. Consequently, similar surgical approaches, autogenous grafts, and polymer-based substitutes are used across these indications.

Each setting, however, imposes distinct requirements on the graft or substitute, and these differences are important for material design. Root coverage requires tissue formation and stabilization over a relatively avascular root surface, making integration dependent on vascular supply from the surrounding recipient bed. Phenotype modification is predominantly preventive, and its success depends not only on the initial dimensional gain but also on its long-term stability. Peri-implant augmentation is particularly demanding because the newly formed tissue must establish and maintain a functional seal around a permanently exposed and microbially colonized transmucosal surface.

The peri-implant soft tissue interface differs fundamentally from the periodontal attachment around natural teeth. It consists of a junctional epithelium attached to the implant or abutment surface through hemidesmosomes and an underlying supracrestal connective tissue compartment [3,4]. There is no cementum or periodontal ligament and no insertion of Sharpey’s fibers into the implant surface; connective tissue fibers are predominantly oriented parallel or circumferentially to the implant surface, and vascular supply is derived mainly from supraperiosteal vessels [5]. These structural differences result in a less mechanically anchored soft tissue interface whose stability depends largely on tissue organization, adhesion, and adequate soft tissue dimensions.

Soft tissue thickness is therefore relevant not only to aesthetics but also to the stability and maintenance of the periodontal and peri-implant environment. Increased peri-implant mucosal thickness has been associated with reduced marginal bone remodeling, while an adequate width of keratinized mucosa has been associated with improved plaque control, patient comfort during oral hygiene, and peri-implant tissue health [6,7]. Around natural teeth, a thicker periodontal phenotype may similarly provide greater resistance to recession and greater tolerance to restorative and orthodontic interventions.

Accordingly, the objective of soft tissue augmentation is not simply to occupy a deficient space, but to achieve a stable and durable increase in tissue thickness, keratinized tissue dimensions, or volume. Autogenous connective tissue and free gingival grafts remain the clinical reference standards because they reliably achieve these objectives across multiple indications. Their disadvantages include a second surgical site, increased operating time, postoperative morbidity, and limited donor availability [8,9,10]. Polymer-based substitutes were developed to reduce these limitations and have substantially improved the morbidity profile of soft tissue augmentation. However, comparative clinical studies frequently report smaller dimensional gains than those achieved with autogenous grafts, particularly for tissue thickness and keratinized tissue width. Understanding the biological and material factors underlying this persistent difference remains a central challenge in the development of next-generation soft tissue substitutes.

### 1.2. Three Categories of Interface Design: Permissive, Bioactive, and Bioresponsive

The materials used to augment oral soft tissues can be grouped into three broad categories of interface design. For the purposes of this review, these categories are distinguished by the direction and degree of information exchange between the material and the host rather than by chemical composition, origin, or clinical indication. This distinction is important because the central argument developed below concerns the transition from materials that merely tolerate or instruct the surrounding tissue to those that modify their behavior in response to local biological conditions.

#### 1.2.1. Biocompatibility as a Prerequisite and the Permissive Scaffold

Biocompatibility is conventionally defined as the ability of a material to perform with an appropriate host response in a specific application [11]. Two features of that definition are consequential. The response must be appropriate rather than absent, since a degree of inflammation is appropriate in a healing wound and inappropriate around a device intended to remain for years; and the definition is qualified by application, because biocompatibility is a property of a material–host–application system rather than of a substance. No implanted material is truly inert: plasma proteins adsorb within seconds and are progressively displaced by others of higher affinity, so that arriving cells encounter a protein layer whose composition the surface has selected. Where no productive interaction follows, the default outcome is a foreign-body reaction proceeding through macrophage recruitment, giant-cell fusion and fibrous encapsulation [12]. For soft tissue augmentation, this is a specific mode of failure, since an encapsulated matrix occupies space without becoming tissue and the volume it appears to provide is lost when it resorbs. Biocompatibility in this sense is a prerequisite shared by all three categories rather than a distinguishing feature of the first. The first category is therefore better described as permissive: a biocompatible scaffold that provides space and a substrate for host colonization without carrying a programmed biological instruction.

#### 1.2.2. Bioactivity

A bioactive material is designed to elicit or modulate a specific biological activity [13]. Historically, the term was used more narrowly to describe materials capable of inducing a defined interfacial response leading to bonding with tissue [14,15]. In contemporary biomaterials science, its meaning has broadened to include materials that actively influence cell adhesion, migration, differentiation, matrix deposition, inflammatory behavior, or antimicrobial activity.

In soft tissue augmentation, bioactivity may arise from surface architecture and chemistry, including fiber organization, porosity, pore interconnectivity, surface texture, and wettability, or from incorporated biological signals such as growth factors, cell-adhesive peptides, extracellular-matrix components, and autologous concentrates [16]. The defining feature is that the material provides a biological instruction that has been established before implantation.

This distinction is important. A material may actively promote fibroblast migration, angiogenesis, or epithelial attachment while still behaving according to a predetermined program. Its output is governed by properties such as adsorption, diffusion, affinity, degradation, or covalent immobilization and does not necessarily change according to whether the surrounding tissue still requires that signal. Such materials can be described as pre-programmed: their release kinetics and degradation profile are governed by properties selected at manufacture. Local tissue conditions do influence them—collagen degradation depends on host proteolytic activity, and affinity-based release follows the local binding equilibrium—but this influence is incidental to the design rather than the product of a deliberately engineered response mechanism [17].

Bone biomaterials have an established vocabulary distinguishing osteoconduction from osteoinduction. No similarly standardized terminology exists for oral soft tissue, and this creates ambiguity: a matrix that merely permits fibroblast infiltration and one that actively recruits or instructs fibroblasts may both be described as “bioactive”. A clearer distinction between permissive scaffolds and instructive materials would therefore improve both material classification and interpretation of clinical evidence.

#### 1.2.3. Bioresponsiveness and Biointeractivity

A bioresponsive material is sensitive to a biological, chemical, or physical signal in its environment and undergoes a functional change in response to that signal [17,18]. Unlike a conventional bioactive material, its behavior is therefore conditional rather than entirely predetermined. A responsive system combines a sensing mechanism with a physicochemical response that may include cleavage, swelling, deswelling, charge reversal, dissolution, conformational change, or altered molecular release.

For the purposes of this review, “biointeractive” is used as a broader functional concept describing bidirectional interaction between the material and the biological environment, whereas “bioresponsive” refers specifically to materials whose behavior changes in response to a measurable local stimulus. This distinction avoids equating responsiveness with full autonomy.

Several characteristics determine the degree of responsiveness of such systems:

1. Recognition specificity—the response is triggered by a biologically relevant signal rather than simply by elapsed time [18].
2. Transduction—detection of the stimulus produces a physicochemical change with a functional consequence, such as bond cleavage, swelling, charge reversal, or degradation [18,19].
3. Threshold behavior—ideally, actuation occurs above a defined stimulus intensity, reducing activation by normal physiological fluctuations [20].
4. Reversibility or switchability—more advanced systems may cease or repeat their response as the stimulus changes rather than exhausting their function in a single discharge [19].
5. Feedback regulation—in the most advanced systems, the material modifies the condition that triggered its response, thereby moving the local environment toward homeostasis [21].

These properties should not be regarded as mandatory features of every bioresponsive material. In particular, responsiveness does not necessarily imply reversibility, feedback control, or autonomy. Classifications of smart biomaterials commonly distinguish active, responsive, and autonomous systems, with true closed-loop self-regulation representing a higher level of functionality than stimulus responsiveness alone [22].

A further distinction is also important. Host-mediated degradation is not automatically equivalent to designed responsiveness. A collagen scaffold degraded by endogenous collagenases is biologically coupled to its environment, but unless the degradation is engineered to respond selectively to a defined biological state, it does not constitute a designed trigger-and-response mechanism. Likewise, the classical concept of “third-generation biomaterials”, referring to molecularly tailored materials designed to stimulate specific cellular responses and gene expression [15], remains fundamentally instructive if the biological program is established before implantation rather than regulated by a built-in response mechanism.

The progression considered in this review can therefore be summarized as follows: permissive materials are tolerated by the host and colonized by whatever it supplies; bioactive materials actively influence the host through pre-programmed signals; and bioresponsive materials modify their behavior according to the state of the surrounding environment (Table 1). Fully autonomous, feedback-controlled systems represent a further level for which no clinical demonstration in oral soft tissue augmentation was identified.

This framework provides the basis for the subsequent analysis of whether the persistent limitations of current soft tissue substitutes can be addressed by improving passive scaffold design alone or whether material behavior that changes with the state of the wound may offer an additional design strategy.

### 1.3. Growth Factors, Carrier-Dependent Delivery Platforms, and the Limitations of Current Platforms

Growth factors represent a rational therapeutic target because they regulate several processes that determine successful scaffold integration and tissue formation. The accumulation of new extracellular matrix depends on colonization of the scaffold by host cells and their subsequent matrix deposition. Several processes that may limit this response are directly regulated by growth-factor signaling [28,29]. Angiogenic ingrowth determines how extensively an implanted matrix can be vascularized and populated [30]; fibroblast recruitment and extracellular matrix synthesis influence what replaces the scaffold during degradation [31]; and keratinocyte migration contributes to epithelial closure and, at transmucosal implant sites, to the establishment of the epithelial component of the soft tissue seal [3,32]. Consequently, loading matrices with platelet-derived growth factor, vascular endothelial growth factor, fibroblast growth factor, or autologous platelet concentrates represents a biologically plausible strategy for enhancing selected phases of soft tissue healing [33,34,35].

The molecular signals governing mucosal repair are well characterized and include distinct regulators of vascular, connective-tissue, and epithelial healing. Platelet-derived growth factor promotes migration and wound repopulation by both gingival and periodontal ligament fibroblasts and drives their early recruitment [34,36,37]. Among its isoforms, the BB homodimer is the most potent mitogen and chemoattractant for human periodontal ligament cells in vitro, with AB intermediate and AA least effective, which underlies the selection of rhPDGF-BB for clinical use [38]. Its mitogenic effect is, however, cell-specific: in an in vitro wound model, periodontal ligament cells proliferated markedly in response to PDGF-BB, whereas gingival fibroblasts showed no proliferative increase at any concentration tested, despite comparable wound-fill responses in both populations [37]. Transforming growth factor-β1 promotes extracellular matrix synthesis but may also drive differentiation of gingival fibroblasts into α-smooth muscle actin-expressing myofibroblasts, contributing to fibrosis and wound contraction [39], whereas TGF-β3 has been associated with a more anti-fibrotic healing phenotype [40]. Oral wounds exhibit lower TGF-β1 levels and a higher TGF-β3/TGF-β1 ratio than cutaneous wounds, a difference that may contribute to the reduced scarring characteristic of oral mucosa [41]. Vascular endothelial growth factor and fibroblast growth factor-2 regulate angiogenic ingrowth [28,42], while keratinocyte growth factor and epidermal growth factor contribute to epithelial migration and repair [28,43]. The biological requirements of oral soft tissue healing are therefore temporally and spatially heterogeneous rather than governed by a single dominant signal.

The principal challenge, however, is not only which biological signal is delivered, but how it is delivered. Free growth factors have limited therapeutic persistence because of their short effective half-lives, susceptibility to proteolytic degradation, and rapid diffusion and dilution within the wound environment [44,45]. These factors reduce local bioavailability and may partly explain the modest and variable effects observed after simple topical administration [44,46]. Effective delivery therefore generally requires association with a carrier through adsorption, entrapment, affinity binding, or covalent immobilization, each of which influences the amount, spatial distribution, and duration of factor availability [46,47].

Current biological delivery approaches can be grouped into several broad platform classes. Xenogeneic and allogeneic matrices provide a resorbable three-dimensional scaffold and rely predominantly on host-derived biological signals [24]. Hydrogels, particularly hyaluronic acid-based systems, combine injectability with intrinsic biological activity and can also function as delivery vehicles [25,48]. Autologous platelet concentrates provide a patient-derived, multifactorial biological payload released from a fibrin network over a limited period [35]. Recombinant growth factors provide a defined molecular signal, while enamel matrix derivative represents a clinically established bioactive protein complex rather than a growth factor itself. These platforms differ substantially in payload composition, carrier properties, release kinetics, standardization, and duration of biological activity.

The resulting biological effect should therefore be considered a property of the bioactive agent-carrier system rather than of the molecule alone. Protein delivery from passive scaffolds is frequently characterized by an initial burst followed by a slower phase governed by diffusion, binding affinity, and carrier degradation [46,47,49]. Depending on the system, this release may occur over a substantially shorter period than the cellular infiltration, extracellular matrix deposition, and tissue remodeling that continue for weeks to months. Likewise, scaffold degradation follows kinetics determined by material composition, architecture, crosslinking, and the local biological environment and is not necessarily synchronized with the formation and maturation of replacement tissue.

Against these platforms stands the autogenous connective tissue graft, which remains the clinical reference standard [8,9]. Its advantage is not simply its collagen composition but its biological constitution. Unlike an acellular substitute, the graft contains living cells and an organized extracellular matrix, becomes revascularized after transplantation, integrates with the recipient tissue, and subsequently participates in ongoing remodeling. Its disadvantages—a second surgical site, increased operating time, postoperative morbidity, and limited availability [9,10]—are precisely those that polymer-based substitutes were designed to reduce.

What current substitutes have not consistently achieved is equivalent long-term gain in tissue thickness and keratinized tissue width; root-coverage outcomes at natural teeth are material- and protocol-dependent. Physiological healing is temporally sequenced, concentration-dependent, and influenced by patient- and site-specific conditions, whereas most currently available platforms release their payload with kinetics determined by the carrier rather than by the state of the wound; local conditions such as proteolytic activity modulate these kinetics, but no mechanism links release to a defined biological need. Several limitations follow from this mismatch. First, the biologically active release window may be shorter than the tissue-remodeling process it is intended to influence. Second, material degradation is not necessarily coupled to the extent of cellular and vascular colonization. Third, once implanted, the timing and magnitude of delivery generally cannot be adjusted to changes in the wound environment. Finally, the effective dose delivered from soak-loaded matrices or autologous concentrates is often incompletely characterized and may vary substantially between preparations.

These limitations do not imply that growth factors or other biological agents are ineffective. Rather, they indicate that their therapeutic performance may depend as much on the carrier, release kinetics, and timing of delivery as on the biological potency of the signal itself. Current bioactive materials can provide increasingly sophisticated biological instructions, but these instructions remain largely determined before implantation. Whether bioresponsive material behavior could better align scaffold persistence and biological signaling with the evolving requirements of soft tissue healing is therefore the central question developed in the subsequent sections of this review.

### 1.4. Aim and Scope

Current polymer-based materials for oral soft tissue augmentation range from passive scaffolds to increasingly bioactive systems capable of supporting cellular colonization or delivering biological signals. However, most currently available platforms lack a deliberately engineered response mechanism: their degradation, mechanical behavior, and release kinetics are set by material design and are modulated by local conditions only incidentally—for example through host proteolytic activity or binding equilibria—without a mechanism that couples them to a specific biological need. Whether this absence of an engineered response contributes to the persistently smaller gains in mucosal thickness and keratinized tissue width observed with soft tissue substitutes compared with autogenous grafts remains unresolved.

The aim of this narrative review is therefore to critically examine the biological and material limitations of current polymer-based substitutes for oral soft tissue augmentation and to assess whether bioresponsive material strategies could address limitations that cannot be overcome by passive scaffold optimization alone. Particular attention is given to the relationship between scaffold architecture, degradation, cellular colonization, growth-factor delivery, and the temporal requirements of mucosal healing.

This review considers three interconnected levels of evidence: (i) the biological requirements of oral soft tissue healing; (ii) the performance and limitations of currently available polymer-based scaffolds and biofunctionalization strategies; and (iii) emerging bioresponsive approaches capable of coupling material behavior to local biochemical, enzymatic, microbial, or mechanical signals. Clinical evidence is used to define the limitations of existing substitutes, while preclinical and materials-science studies are evaluated to identify potential strategies for overcoming them.

Particular emphasis is placed on the concept that the effectiveness of biological augmentation depends on the interaction between the bioactive agent and the carrier rather than on either component in isolation. This review also examines cell-seeded scaffolds as a possible means of introducing environmentally responsive behavior through living cells, while recognizing their distinct manufacturing, regulatory, and translational requirements.

Literature search. A structured narrative literature search was performed in PubMed/MEDLINE, Scopus, Web of Science Core Collection, and the Cochrane Library for English-language publications from 1 January 2000 to July 2026, when the last search update was performed; the exact day of the final search was not recorded. Four search strings were combined with the Boolean operator AND, with syntax and search fields adapted to each database: (1) clinical setting—(“soft tissue augmentation” OR “gingival recession” OR “root coverage” OR “keratinized tissue” OR “keratinized mucosa” OR “mucosal thickness” OR “peri-implant soft tissue” OR “peri-implant mucosa”); (2) material—(“connective tissue graft” OR “collagen matrix” OR “xenogeneic collagen” OR “acellular dermal matrix” OR “hyaluronic acid” OR hydrogel OR scaffold OR polymer); (3) biological functionalization—(“platelet-rich fibrin” OR “platelet concentrate” OR “growth factor” OR rhPDGF OR “enamel matrix derivative” OR “gingival fibroblast” OR “cell seeding” OR “cell-seeded”); and (4) responsive materials—(“smart polymer” OR “stimuli-responsive” OR “stimulus-responsive” OR bioresponsive OR “enzyme-responsive” OR “pH-responsive” OR “MMP-cleavable” OR “gingipain-responsive”). Strings (1) AND (2), (1) AND (3), and (2) AND (4) were run separately, and the reference lists of included reviews were screened manually for additional sources.

Records were included if they were original clinical studies, systematic reviews, meta-analyses, or consensus reports on soft tissue augmentation around teeth or implants, or preclinical and materials-science studies describing a polymer system with demonstrated or explicitly proposed relevance to oral soft tissue healing. Case reports, conference abstracts without full text, and studies restricted to bone regeneration, orthopedic applications, or non-oral soft tissues were excluded unless they provided transferable mechanistic evidence. Foundational publications predating 2000 and selected studies from other tissue-engineering applications were additionally included when they informed the conceptual framework or provided transferable mechanistic evidence. Titles and abstracts were screened by N.M., and full texts of potentially eligible records were assessed by both authors, with disagreement resolved by discussion. As is usual for a narrative review, no formal record of the number of retrieved and excluded records was kept; the reference list comprises 180 sources, including original studies, systematic reviews, consensus reports, standards, and methodological references, and therefore does not represent a count of studies qualifying after screening. Because this review is narrative, the search was structured but not exhaustive: where several studies supported the same point, the strongest available clinical evidence, the most informative preclinical study, or the most recent comprehensive review was selected. Statements that no clinical evidence exists for a given strategy therefore refer to the literature identified by this search and not to an exhaustive systematic synthesis.

The overarching objective is to define which limitations of current soft tissue substitutes are primarily biological, which are material-dependent, and which may require a transition from passive or instructive scaffolds toward systems capable of responding dynamically to the local tissue environment.

## 2. Biology of Soft Tissue Healing and Implications for Polymer Design

Successful tissue engineering requires not only appropriate cell populations and scaffold properties but also a material environment capable of supporting the sequence of biological events through which new tissue forms and matures [50]. Cells continuously interact with soluble mediators, neighboring cells, and the extracellular matrix, creating a dynamic microenvironment that regulates migration, proliferation, differentiation, and matrix production [51]. For polymer-based soft tissue substitutes, the relevant design problem is therefore not simply to provide a biocompatible space, but to support a healing process whose biological requirements change over time [46,52].

### 2.1. The Mucosal Healing Cascade as a Timed Sequence

**Figure 1:**
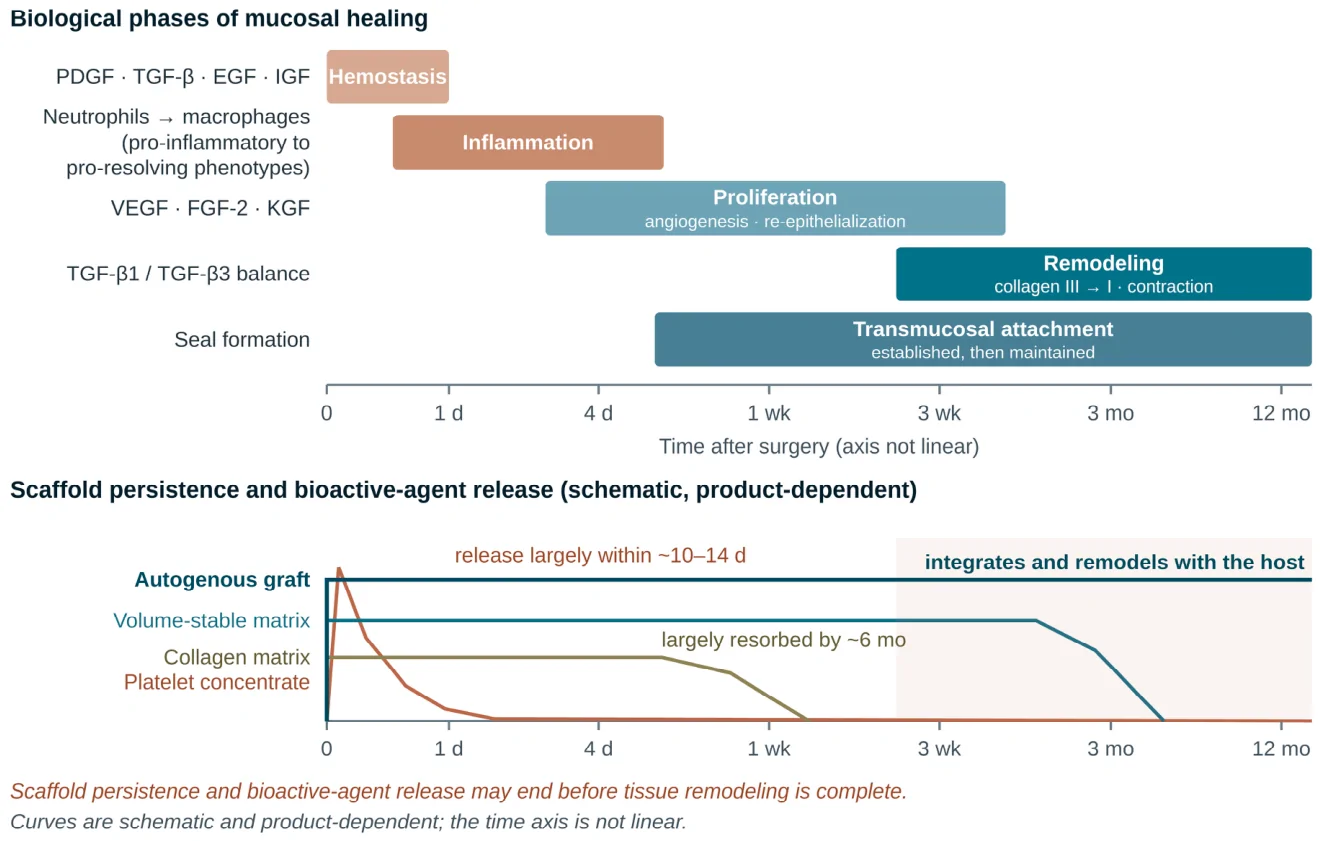
Mucosal healing timeline showing the dominant biological processes during hemostasis, inflammation, proliferation, and remodeling (upper panel), and the approximate persistence or bioactive-agent release windows of platelet concentrates, collagen matrices, volume-stable matrices, and autogenous grafts (lower panel). Curves are schematic and product-dependent, and the time axis is not linear. Scaffold persistence and bioactive-agent release may end before tissue remodeling is complete; this does not imply that tissue formed with a substitute stops remodeling. EGF, epidermal growth factor; FGF-2, fibroblast growth factor-2; IGF, insulin-like growth factor; KGF, keratinocyte growth factor; PDGF, platelet-derived growth factor; TGF-β, transforming growth factor-beta; VEGF, vascular endothelial growth factor. Phase durations after references [53,54].

Mucosal repair proceeds through four overlapping phases—hemostasis, inflammation, proliferation, and remodeling—each characterized by distinct cellular populations, mediators, and structural requirements [53,54]. Because the biological demands placed on an implanted scaffold differ substantially between these phases, the healing timeline provides a useful framework for defining material requirements (Figure 1). The following sections therefore consider each phase together with its principal implications for scaffold design.

#### 2.1.1. Coagulation and Hemostasis (Minutes to 1 Day)

Injury to the gingival or mucosal vasculature initiates transient vasoconstriction, platelet adhesion and activation, and the coagulation cascade, resulting in formation of a fibrin-rich provisional matrix [54]. Platelets bind exposed extracellular matrix components, become activated, and release a broad range of signaling molecules, including platelet-derived growth factor (PDGF), transforming growth factor-β (TGF-β), epidermal growth factor (EGF), and insulin-like growth factor (IGF) [53,55]. Although release is greatest during the early post-injury period, activated platelets may continue to secrete mediators for several days [55].

Material implication. The earliest requirement for an implanted scaffold is the establishment of a stable blood clot and a continuous interface with the recipient bed. Surface chemistry, wettability, architecture, and blood absorption therefore influence the provisional matrix on which subsequent cellular infiltration depends. At this stage, the material must support rather than disrupt hemostasis and early cell attachment.

#### 2.1.2. Inflammation (Hours to Approximately 4 Days)

The inflammatory phase begins rapidly through damage-associated molecular signals and chemokine gradients that recruit neutrophils and subsequently monocytes and macrophages [53,54,56]. Neutrophils provide early antimicrobial defense through phagocytosis, degranulation, and extracellular trap formation, while also releasing proteases, including matrix metalloproteinases (MMPs), that remodel the provisional extracellular matrix. Excessive or prolonged neutrophil activity, however, can degrade extracellular matrix components, growth factors, and their receptors and thereby interfere with subsequent repair.

Monocytes subsequently differentiate into macrophages, which coordinate both inflammatory activity and its resolution [57,58]. The transition from predominantly pro-inflammatory to reparative macrophage states is a key step in the progression from inflammation to tissue formation [53,59]. Resolution is an active process involving changes in lipid mediators and clearance of inflammatory cells rather than simple disappearance of the inflammatory response [53]. Material implication. An implanted scaffold must permit an appropriate transient inflammatory response without promoting its persistence. Material composition, degradation products, residual crosslinking agents, surface characteristics, and degradation rate can all influence this balance. A prolonged foreign-body or inflammatory response may impair vascularization and cellular colonization irrespective of any bioactive agent incorporated within the scaffold.

#### 2.1.3. Proliferation and Granulation Tissue Formation (Approximately 3 Days to 3 Weeks)

Three interrelated processes dominate this phase: angiogenesis, fibroblast-mediated matrix formation, and re-epithelialization.

*Angiogenesis*

Newly forming vessels grow into the wound in response to local hypoxia and angiogenic signaling, particularly VEGF [54,60,61]. Vascular ingrowth is critical for scaffold integration because it determines oxygen and nutrient delivery to cells migrating into the material.

*Fibroblast recruitment and extracellular matrix deposition*

Gingival fibroblasts migrate into the provisional matrix in response to PDGF, TGF-β, IGF-1, and extracellular matrix cues and begin synthesizing collagen, fibronectin, proteoglycans, and hyaluronan [53]. A proportion of these cells differentiate into α-smooth muscle actin-positive myofibroblasts under the combined influence of TGF-β1, matrix composition, and mechanical stiffness [54,62]. Myofibroblasts generate contractile forces that contribute to wound closure but may simultaneously reduce the volume of newly formed tissue [63,64].

*Re-epithelialization*

Keratinocytes migrate across the provisional matrix, remodel the extracellular environment through proteolytic enzymes, and subsequently re-establish basement membrane attachment and epithelial differentiation [53,54,65]. Material implication. This phase creates one of the central design conflicts in soft tissue augmentation. The scaffold must be sufficiently porous and interconnected to permit vascular and fibroblast infiltration, while retaining enough mechanical stability to resist compression and contraction during tissue formation. If cellular and vascular invasion does not progress throughout the scaffold before substantial degradation occurs, part of the implanted volume may be lost without equivalent replacement by newly formed tissue. At the same time, the biological process responsible for populating the scaffold also generates contractile forces that may reduce the volume the procedure was intended to create.

#### 2.1.4. Remodeling and Maturation (Approximately 3 Weeks to 12 Months and Beyond)

During remodeling, inflammatory cells, excess endothelial cells, and myofibroblasts progressively decline, while the provisional extracellular matrix is reorganized. Type III collagen characteristic of early granulation tissue is gradually replaced by more mature type I collagen, and the balance between MMPs and tissue inhibitors of metalloproteinases regulates matrix turnover [53,66,67]. Vascular density also decreases as the initially abundant neovasculature matures into a more stable network [54]. Material Implication. Remodeling extends substantially beyond the early period of scaffold implantation and biological-agent release. The long-term dimensional outcome therefore depends on how closely scaffold degradation is matched by the formation and maturation of replacement tissue. Passive materials may persist for different periods depending on their composition and crosslinking, but their degradation kinetics are generally not designed to change according to the degree of tissue maturation. This temporal relationship between scaffold loss and tissue replacement is particularly relevant to the long-term stability of soft tissue augmentation.

#### 2.1.5. Establishment and Maintenance of the Transmucosal Attachment

Where a dental implant or abutment is present, wound healing includes an additional requirement beyond conventional closure. Junctional epithelium forms an attachment to the transmucosal surface through hemidesmosomes and an internal basal lamina, while a supracrestal connective tissue compartment organizes beneath it. Because this connective tissue lacks the inserting fiber architecture characteristic of the periodontal ligament, the peri-implant barrier depends strongly on epithelial attachment, connective tissue organization, and soft tissue dimensions [68].

Unlike wound closure, which has a definable healing period, maintenance of the transmucosal barrier is a long-term requirement. This distinction is particularly relevant to material design because a temporary scaffold or single-release biological intervention may support initial healing without necessarily addressing the conditions required for long-term barrier stability.

### 2.2. Distinctive Features of Oral Mucosa Healing

Oral mucosal wounds generally heal more rapidly and with less scar formation than comparable cutaneous wounds. This has been associated with a reduced inflammatory response, differences in TGF-β isoform expression, distinctive properties of gingival fibroblasts, and the presence of saliva-derived factors, including histatins and antimicrobial peptides [53,69].

These characteristics are relevant to biomaterial design because oral soft tissue should not be considered simply a smaller version of cutaneous connective tissue. A scaffold intended for this environment must interact with a cell population and signaling profile that already possess substantial regenerative potential. An important future direction may therefore be to design materials that preserve or support the favorable phenotype of oral mucosal cells rather than merely providing physical space for their infiltration. Strategies that modulate the balance between profibrotic and antifibrotic signaling, or that maintain the regenerative phenotype of gingival fibroblasts, represent examples of this tissue-specific approach.

### 2.3. Biological Constraints on Carrier Design

The healing cascade highlights three properties that any biologically active carrier must accommodate.

First, healing is temporally sequenced. Different mediators predominate at different stages, and a signal beneficial during one phase may be unnecessary or potentially detrimental when sustained beyond its physiological window. For example, prolonged TGF-β1 signaling may favor myofibroblast differentiation and fibrosis rather than regenerative remodeling.

Second, signaling is concentration-dependent and actively regulated. Physiological growth-factor activity is controlled through synthesis, binding, proteolytic degradation, receptor availability, and endogenous inhibitors. An effective delivery system therefore needs to provide not merely sustained release, but biologically appropriate exposure within a relevant concentration range.

Third, healing is patient- and site-dependent. Age, smoking, systemic conditions, local vascularity, tissue phenotype, and inflammatory burden may alter healing kinetics and the composition of patient-derived biological preparations [53]. A release profile optimized for an average healing response may therefore be suboptimal in sites or patients whose biological sequence is delayed or altered.

Together, these constraints demonstrate that duration alone is an incomplete design objective. A carrier may release a biological factor for longer without necessarily releasing it at the appropriate time, concentration, or biological state.

### 2.4. The Native Extracellular Matrix as a Model for Regulated Signal Availability

The extracellular matrix is not merely a structural substrate for cells; it also regulates the availability of biological signals. Growth factors can bind to heparan sulfate proteoglycans within the extracellular matrix and subsequently be released or redistributed through enzymatic modification of these binding sites [70]. The matrix therefore functions as a dynamic reservoir that influences when and where signaling molecules become available.

Mechanical regulation provides a further example. During wound healing, integrin-mediated cellular forces can contribute to activation of latent TGF-β1 stored within the extracellular matrix, linking local mechanical conditions to biochemical signaling [65,71]. Such mechanisms illustrate how native tissues couple matrix state, cellular activity, and signal availability rather than treating them as independent processes.

For biomaterial design, this provides an important conceptual model: the objective need not simply be prolonged release but release that is coupled to biologically relevant changes in the local environment. Synthetic and natural polymer systems that reproduce selected aspects of this regulation form the basis for the bioresponsive strategies discussed later in this review.

## 3. Polymer-Based Platforms for Oral Soft Tissue Augmentation

Polymer-based materials used for oral soft tissue augmentation differ substantially in their origin, architecture, degradation mechanism, mechanical properties, and biological behavior. These differences determine not only how long a scaffold occupies the augmented space, but also how rapidly it becomes vascularized and colonized, how it responds to mechanical loading, and whether degradation is matched by formation of replacement tissue. The following section considers the major material classes currently used or investigated for soft tissue augmentation, using autogenous tissue as the biological reference against which their performance can be interpreted [7].

### 3.1. Autogenous Tissue as the Biological Reference Standard

The subepithelial connective tissue graft and free gingival graft are not polymer-based substitutes, but their biological properties define the performance target that engineered scaffolds attempt to reproduce. Autogenous grafts contain a native extracellular matrix with hierarchical collagen organization together with resident cells and vascular structures capable of revascularization and subsequent remodeling within the recipient site. In contrast to resorbable substitutes, they do not have a predetermined degradation endpoint but become incorporated into tissue that continues to remodel with the host.

The connective tissue graft remains the reference approach for increasing soft tissue thickness and achieving root coverage, whereas the free gingival graft remains particularly effective for increasing keratinized tissue width [2,72]. Their principal limitations are well established: a second surgical site, postoperative morbidity, increased operating time, finite donor availability, and, particularly for free gingival grafts, potential color and texture mismatch [73].

From a materials perspective, autogenous grafts therefore provide an important benchmark not because they represent an ideal scaffold composition, but because they combine architecture, cellularity, vascular integration, and long-term remodeling within a single living construct. No currently available off-the-shelf substitute reproduces all of these features simultaneously.

### 3.2. Xenogeneic Collagen Matrices

Xenogeneic collagen matrices, most commonly derived from porcine dermis or other collagen-rich tissues, are among the most extensively investigated substitutes for autogenous connective tissue. Their principal advantages include unlimited availability, avoidance of donor-site surgery, favorable handling, and an extracellular-matrix composition that supports adhesion and migration of host cells [24,74].

Their performance depends strongly on collagen source, processing, fiber architecture, porosity, pore interconnectivity, thickness, and the preservation or removal of native extracellular-matrix components. These characteristics influence hydration, mechanical behavior, cellular infiltration, vascular penetration, and enzymatic degradation.

Non-crosslinked collagen matrices are degraded predominantly through host-mediated enzymatic processes. Although this makes degradation biologically influenced, the rate is not necessarily coordinated with the extent of vascular or cellular replacement of the scaffold. Substantial remodeling and loss of the original collagen structure may occur within the first months after implantation [75].

The major clinical limitation is therefore dimensional loss during remodeling. A scaffold must retain sufficient volume long enough to permit replacement by newly formed connective tissue, but prolonging persistence by modifying collagen chemistry may itself alter tissue integration. This balance between rapid biological integration and sufficient volumetric stability is a recurring design problem across collagen-based substitutes.

### 3.3. Volume-Stable and Crosslinked Collagen Matrices

Crosslinking was introduced primarily to reduce rapid degradation and improve mechanical and volumetric stability. Intermolecular bonds between collagen fibers increase resistance to enzymatic cleavage, allowing the scaffold to persist longer under physiological conditions [76].

The effect of crosslinking, however, is not simply to extend degradation time. Crosslink density and chemistry also influence stiffness, swelling, pore architecture, cellular migration, vascular penetration, and the inflammatory or foreign-body response. Consequently, increasing resistance to degradation can create a trade-off between volume persistence and biological integration.

Volume-stable collagen matrices exploit this principle by combining crosslinked collagen with an architecture designed to resist collapse under flap pressure and wound contraction. They can provide improved maintenance of space compared with rapidly resorbing matrices, but clinical comparisons indicate that prolonged persistence does not necessarily result in equivalent thickness gain compared with autogenous connective tissue [7,76].

Crosslinking should be regarded as a means of shifting the degradation trajectory rather than solving the underlying coordination problem between scaffold persistence and tissue formation. The material persists for a longer period set by the degree of crosslinking and the local proteolytic activity, but it does not persist specifically until sufficient replacement tissue has formed.

### 3.4. Acellular Dermal Matrices

Acellular dermal matrices are derived from allogeneic or xenogeneic dermal tissues processed to remove cellular and antigenic components while retaining much of the native extracellular-matrix architecture. Their collagen network and residual basement-membrane components provide a biologically recognizable substrate for host–cell migration and tissue integration.

Clinical evidence indicates that acellular dermal matrices can achieve root-coverage outcomes approaching those obtained with autogenous connective tissue in selected indications, although increases in keratinized tissue dimensions may remain smaller [77].

Their material behavior is highly dependent on processing. Decellularization, sterilization, preservation, crosslinking, and tissue source may modify collagen ultrastructure [78,79], mechanical strength, porosity, surface chemistry, and degradation rate. These processes may also introduce batch-to-batch variability that is not evident from the generic term “acellular dermal matrix”.

Accordingly, the biological identity of the source tissue does not by itself define scaffold performance; processing history becomes part of the material. This is particularly relevant when comparing different commercial products or when such matrices are used as carriers for growth factors or cells.

### 3.5. Synthetic Biodegradable Polymers

Synthetic biodegradable polymers provide a fundamentally different design space because their physicochemical properties can be controlled more precisely during fabrication. Poly(lactic-co-glycolic acid) (PLGA), polylactic acid (PLA), polycaprolactone (PCL), and poly(L-lactide-co-caprolactone) (PLCL) can be manufactured as electrospun meshes, porous scaffolds, membranes, or three-dimensional printed constructs and have been investigated primarily as barrier materials or components of composite regenerative systems [80,81].

Their degradation can be modified through molecular weight, copolymer composition, crystallinity, hydrophilicity, geometry, pore structure, and scaffold thickness. In PLGA, for example, altering the lactide-to-glycolide ratio changes water uptake and hydrolysis, while the higher crystallinity and hydrophobicity of PCL permit substantially slower degradation.

This degree of tunability represents an important advantage over naturally derived matrices. Nevertheless, three limitations remain relevant to oral soft tissue applications. First, hydrolytic degradation of aliphatic polyesters may generate acidic products, potentially altering the local microenvironment. Second, processing methods involving organic solvents, heat, or other fabrication conditions may complicate incorporation of labile proteins or living cells. Third, tunability remains predominantly pre-implantation tunability: the degradation trajectory is set during manufacture and, beyond the incidental influence of local conditions on hydrolysis, is not coupled to the biological progress of healing.

Synthetic polymers therefore offer superior control over material parameters but not necessarily adaptive control after implantation. Their greatest potential may lie in composite or responsive systems in which this manufacturing precision is combined with biologically triggered behavior.

### 3.6. Hydrogels and Injectable Polymer Systems

Hydrogels provide a complementary strategy to preformed matrices. Their highly hydrated three-dimensional polymer networks can conform to irregular anatomical spaces and can serve simultaneously as fillers, cell carriers, and delivery systems for biological agents.

Hyaluronic acid is particularly relevant to oral soft tissue healing because it is a natural extracellular-matrix component involved in hydration, cell migration, angiogenesis, and inflammatory regulation [25]. Other investigated hydrogel-forming polymers include chitosan and synthetic polyethylene glycol-based networks. Depending on composition, gelation can be achieved through ionic interactions, chemical crosslinking, temperature-dependent transitions, or photopolymerization.

The principal advantage of hydrogels is spatial conformity and chemical versatility rather than mechanical space maintenance. They can reach papillary or irregular defects that are difficult to fill with a preformed scaffold and can be engineered to incorporate growth factors, peptides, extracellular vesicles, or cells.

Their principal limitation in conventional form is mechanical. Many hydrogels have insufficient stiffness and resistance to compression to maintain augmented volume beneath a mobilized flap and may undergo relatively rapid degradation or dissolution. For soft tissue augmentation, the challenge is therefore to reconcile injectability and biological permissiveness with sufficient mechanical integrity and persistence.

Hydrogels are also particularly important for the later discussion of bioresponsive materials because changes in crosslink density, swelling, charge, or degradation can be engineered to occur in response to environmental stimuli, making polymer networks well suited to conditional rather than exclusively time-dependent delivery.

### 3.7. Transmucosal Surface Interfaces

Soft tissue augmentation around implants involves two interacting material interfaces: the temporary or resorbable augmentation material within the connective tissue and the permanent implant or abutment surface against which the tissue must establish a seal. The latter has often been considered separately from the scaffold literature, although both influence the same biological compartment.

Abutment composition, surface topography, roughness, surface energy, wettability, and antimicrobial modification influence epithelial and fibroblast attachment at the transmucosal interface [82]. Surface modification strategies have therefore been developed to promote epithelial adhesion, connective tissue organization, and resistance to bacterial colonization.

This distinction is important for the present review because the requirements of the two material classes differ fundamentally. An augmentation scaffold is expected to degrade and be replaced by tissue, whereas a transmucosal implant surface remains permanently present and must support tissue attachment under continuous microbial and mechanical challenge. The former raises the question of how degradation should be coupled to tissue formation; the latter raises the question of how an interface should maintain biological function indefinitely.

These two problems converge in the concept of bioresponsive interfaces discussed later in this review.

### 3.8. Design Variables Governing Passive Matrix Performance

Across the material classes described above, the performance of a passive scaffold is determined by several interdependent variables [83]:

1. Degradation mechanism and rate—natural collagen-based matrices are degraded predominantly through enzymatic mechanisms, whereas many synthetic aliphatic polyesters degrade principally by hydrolysis. The relevant parameter is not simply how rapidly degradation occurs, but how scaffold loss relates temporally and spatially to formation of replacement tissue.
2. Porosity, pore size, and interconnectivity—these parameters determine the movement of fluid, diffusion of nutrients and signaling molecules, cellular penetration, and vascular ingrowth. High porosity may improve biological infiltration but can reduce mechanical strength and volumetric stability, illustrating the interdependence of scaffold properties.
3. Mechanical properties—a soft tissue scaffold must withstand hydration, trimming, flap pressure, suturing forces, wound contraction, and functional loading without collapsing or becoming excessively rigid. Relevant properties include stiffness, resilience, compressive resistance, tensile behavior, and shape recovery.
4. Surface chemistry and wettability—these influence initial protein adsorption, blood interaction, cell adhesion, and subsequent host response. Surface modification can therefore alter biological performance without changing the bulk composition of the scaffold.
5. Architecture and spatial organization—fiber orientation, pore gradients, layered construction, and local differences in stiffness may influence whether different regions of the scaffold preferentially support epithelial, fibroblastic, or vascular responses. A homogeneous material may not optimally serve a biologically heterogeneous interface.
6. Volumetric stability—volumetric stability is not an isolated material parameter but an emergent outcome of degradation, architecture, mechanical properties, cellular infiltration, vascularization, and wound contraction. It therefore cannot be optimized independently of the variables above.

These parameters provide substantial scope for material optimization. Crosslinking can prolong persistence; altered porosity can improve cellular infiltration; surface chemistry can enhance attachment; and synthetic fabrication can generate spatially controlled architectures.

A scaffold can therefore be designed to degrade rapidly or slowly, to be stiff or compliant, porous or dense, and bioactive or relatively inert. What it generally cannot do is modify these properties according to whether the surrounding tissue has reached the biological state for which that change is required.

This distinction between tunability and responsiveness is central to the argument of this review. Tunability describes the ability of the designer to select material behavior before implantation; responsiveness describes the ability of the material to alter that behavior after implantation in response to the local biological environment.

Table 2 summarizes the principal characteristics, advantages, and limitations of the major autogenous and polymer-based platforms used for oral soft tissue augmentation.

## 4. Biofunctionalization Strategies for Soft Tissue Matrices: From Passive Loading to Living and Immobilized Signals

Biofunctionalization aims to extend the role of a scaffold beyond structural support by incorporating biological signals, modifying their presentation, or introducing cells capable of generating such signals. The terminology used to describe these approaches is inconsistent, and strategies that differ substantially in mechanism are often grouped together as “bioactive” matrices. For oral soft tissue augmentation, a more useful classification is based on how the biological signal is associated with the scaffold and how much control the system provides over its availability after implantation.

Five principal strategies can be distinguished: (1) physical adsorption or soak-loading; (2) bioactivation with autologous concentrates; (3) cell-seeded or prevascularized constructs; (4) affinity-based retention; and (5) covalent immobilization. These strategies should not be interpreted as a strict linear hierarchy. Physical adsorption, affinity-based retention, and covalent immobilization can be positioned along a continuum of increasing binding strength, whereas autologous concentrates and living cells differ primarily in the nature of the biological payload. The framework describes increasing sophistication in signal presentation and control rather than five sequential generations of the same technology.

### 4.1. Physical Adsorption and Soak-Loading

The simplest method of biofunctionalization is to immerse a scaffold in, or apply to it, a solution containing the desired biological molecule. Protein retention occurs through non-covalent interactions with the material surface and physical entrapment within pores, without a specifically engineered binding mechanism [44].

This approach has several practical advantages. It requires no additional chemical modification, can preserve the original structure of the scaffold, and is compatible with chairside application. However, the amount of protein retained by the material may differ substantially from the amount initially applied, and both loading efficiency and subsequent release depend on scaffold composition, porosity, surface charge, wettability, and protein-surface affinity [44,87]. Adsorption may also alter protein conformation and thereby reduce the fraction that remains biologically active [87].

Release from physically loaded scaffolds is typically dominated by desorption and diffusion and may therefore show a pronounced early burst followed by rapidly declining availability. The magnitude and duration of this burst vary between materials and proteins and should not be assumed to be identical across systems.

Physical loading provides biological supplementation with minimal manufacturing complexity but limited control overdose, spatial distribution, and release kinetics. Its major translational advantage—simplicity—is also its principal pharmacological limitation.

### 4.2. Bioactivation with Autologous Platelet Concentrates

Autologous platelet concentrates represent a clinically accessible form of scaffold biofunctionalization in which the payload consists not of a single recombinant molecule but of a patient-derived mixture of growth factors, cytokines, extracellular-matrix proteins, and other signaling components.

Platelet-rich fibrin and related preparations can be combined with collagen matrices or membranes by direct contact, impregnation, or incorporation into composite constructs. Experimental studies have demonstrated that combining platelet concentrates with collagen-based matrices can modify the release of VEGF, TGF-β, and other mediators and may enhance angiogenic responses compared with the untreated scaffold [88,89]. Similarly, incorporation of injectable platelet-rich fibrin into collagen-chitosan hydrogels has been reported to prolong the availability of TGF-β1 and PDGF-AB relative to the platelet preparation alone [90].

Importantly, the biological effect is not determined solely by the concentrate. The interaction between the concentrate and the carrier influences retention, release kinetics, and potentially the biological response. This observation supports the broader concept developed in this review that the bioactive agent–carrier pairing constitutes the functional therapeutic system.

The principal advantages of autologous concentrates are chairside availability, autologous origin, and delivery of multiple biological signals without the need for recombinant proteins. Their limitations include patient- and preparation-dependent variability, incomplete standardization of protocols, uncertain effective dose, and predominantly early biological activity. Thus, although platelet concentrates provide a more physiologically complex payload than single-factor loading, their release follows fibrin degradation and cannot be adjusted once the construct has been implanted.

### 4.3. Cell-Seeded and Prevascularized Constructs

Cell-based biofunctionalization represents a fundamentally different strategy because the biological component is living rather than stored and subsequently released. Fibroblasts, keratinocytes, endothelial cells, mesenchymal stromal cells, or combinations of these populations can be seeded onto or within a scaffold before implantation [91,92].

For oral soft tissue augmentation, fibroblast-seeded matrices are particularly relevant because fibroblasts can colonize the scaffold, synthesize extracellular matrix, and respond dynamically to biochemical and mechanical cues within the surrounding environment. Unlike a growth-factor reservoir whose release is set by the carrier, living cells can alter the quantity and composition of signals they produce according to their local conditions. In principle, cell-seeded constructs could therefore introduce a degree of biological responsiveness that passive delivery systems cannot provide. Whether this occurs after implantation depends on the survival and continued function of the transplanted cells, which the clinical studies available to date have not examined directly (Section 8.5).

Prevascularization extends this approach by combining stromal cells with endothelial cells or preformed microvascular structures before implantation [92]. The objective is to shorten the interval during which centrally located cells depend exclusively on diffusion and to facilitate earlier integration with the host vasculature.

Cell seeding should nevertheless not be equated with an engineered polymer that senses and responds to a defined stimulus. Cellular behavior is environmentally adaptive, but it remains influenced by scaffold properties, cell source, phenotype, passage number, seeding density, and local host conditions. Moreover, cell-based products introduce major translational challenges, including cell expansion, sterility, viability, storage and transport, manufacturing reproducibility, cost, and potentially advanced-therapy regulatory classification [93].

The principal conceptual advantage is therefore not simply that cells “replace what the scaffold lacks”, but that they could in principle provide a renewable and environmentally responsive source of extracellular matrix and biological signals, provided that they survive and remain functional after implantation.

### 4.4. Affinity-Based Retention

Affinity-based delivery uses reversible molecular interactions to retain bioactive proteins within a scaffold more effectively than simple physical adsorption. Heparin, heparan sulfate, glycosaminoglycan mimetics, and related functional groups can bind growth factors through naturally occurring affinity domains [94].

This approach partially reproduces the role of the native extracellular matrix, in which glycosaminoglycans act as reservoirs for signaling molecules and influence their local availability [70,95]. Heparin-functionalized and related affinity-based scaffolds can reduce burst release, protect bound proteins from rapid diffusion or degradation, and prolong local availability compared with non-specific adsorption [96,97]. Several mediators relevant to mucosal repair, including VEGF and FGF-2, possess heparin-binding domains and are therefore suitable candidates for this strategy.

Affinity binding provides greater control over retention without converting the biological molecule into a permanently immobilized signal. Release remains reversible and can be influenced by binding affinity, competitive interactions, scaffold degradation, and the local biochemical environment.

However, in most affinity systems, the density and strength of binding sites are established during fabrication, and prolonged retention does not itself ensure that release occurs when the surrounding tissue biologically requires the signal.

Affinity-based delivery reproduces the reservoir function of the native extracellular matrix more closely than simple adsorption but generally does not reproduce its complete regulatory machinery.

### 4.5. Covalent Immobilization

Covalent immobilization represents a different strategy in which a biological ligand is chemically attached to the polymer or scaffold surface. Cell-adhesive peptides derived from fibronectin, laminin, or other extracellular-matrix proteins—including RGD-containing sequences—can be covalently grafted onto biomaterial surfaces to provide stable spatial presentation rather than controlled release [98].

The biological response depends not only on ligand identity but also on surface density, orientation, accessibility, linker length, and spatial organization [99,100]. Gingival and periodontal fibroblasts express integrins capable of interacting with several of these extracellular-matrix motifs [101], making ligand immobilization relevant to oral soft tissue engineering.

The principal advantage is that a covalently bound ligand can remain associated with the material and resist rapid diffusion or mechanical displacement. This can provide prolonged and spatially defined cell-material signaling without the burst release characteristic of soluble-factor delivery.

The limitation is that immobilization transforms a naturally dynamic biological signal into a material property whose availability is set by the carrier rather than regulated by the tissue. Depending on the chemistry used, covalent attachment may alter ligand conformation, orientation, accessibility, or biological function. Moreover, permanent presentation may be undesirable when the physiological requirement for a particular signal is transient.

Covalent immobilization should therefore be considered a strategy for controlling spatial presentation rather than simply the highest level of sustained release. Its usefulness depends on whether persistent signaling is biologically appropriate for the intended function.

### 4.6. What the Biofunctionalization Framework Demonstrates

**Figure 2:**
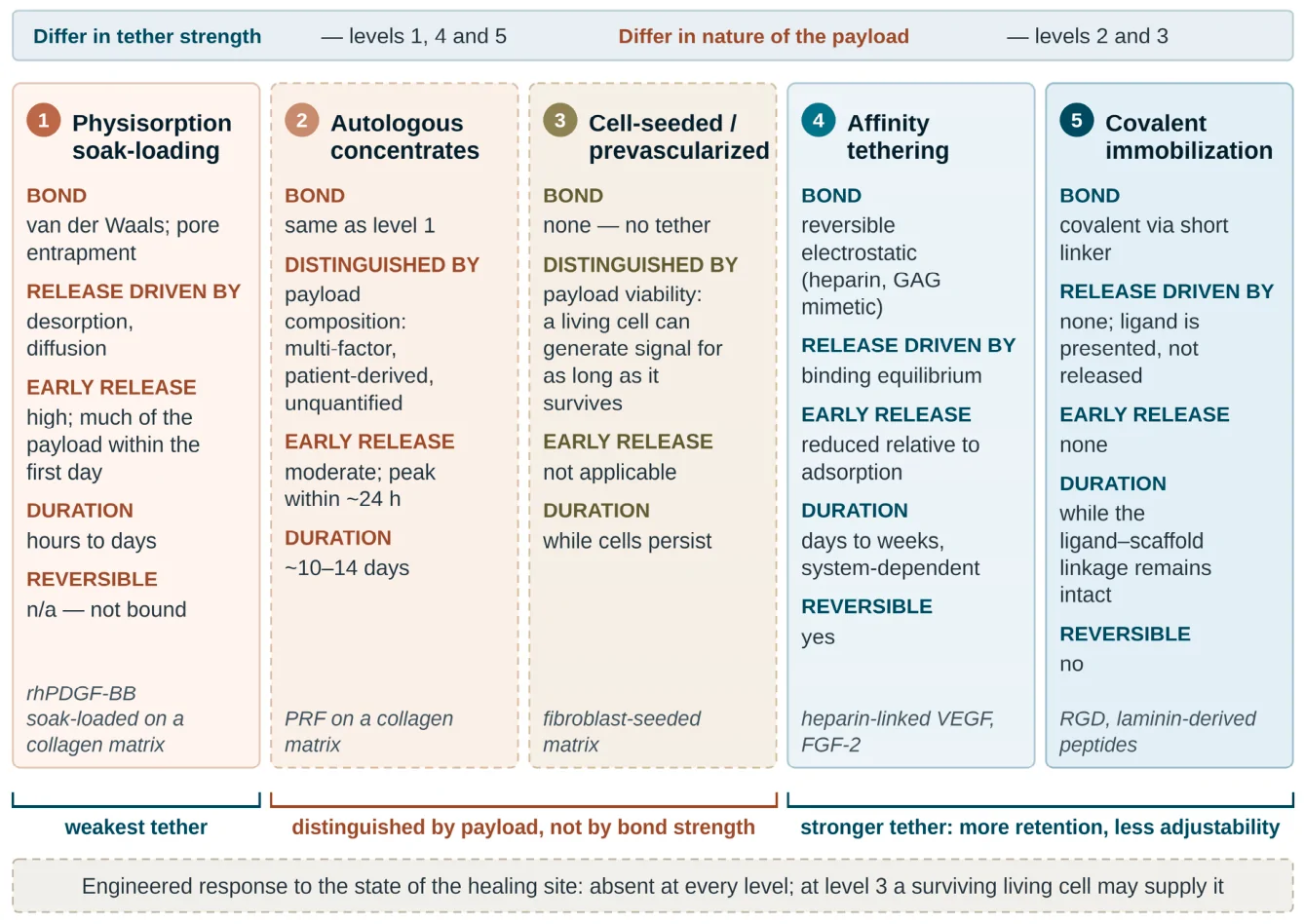
Biofunctionalization strategies for oral soft tissue matrices. Physical adsorption, affinity-based retention, and covalent immobilization (levels 1, 4, and 5) differ in the strength of molecular association with the scaffold, whereas autologous concentrates and cell-seeded constructs (levels 2 and 3) differ in the nature of the payload and are not steps on that scale. Entries are qualitative and system-dependent; representative quantitative data and sources are given in Section 4.1, Section 4.2, Section 4.3, Section 4.4 and Section 4.5 and Table 3. GAG, glycosaminoglycan; FGF-2, fibroblast growth factor-2; PRF, platelet-rich fibrin; RGD, arginine–glycine–aspartic acid motif; rhPDGF-BB, recombinant human platelet-derived growth factor-BB; VEGF, vascular endothelial growth factor.

Considered together, these strategies illustrate three different dimensions of control: retention of the biological signal, duration and spatial presentation, and capacity to adapt to the biological environment (Figure 2, Table 3). Improving one dimension does not necessarily improve the others.

Physical adsorption provides minimal control but maximal simplicity. Affinity-based retention can prolong availability while maintaining reversibility. Covalent immobilization provides stable spatial presentation but limits temporal adaptability. Autologous platelet concentrates deliver multiple physiological signals but with substantial biological variability. Cell-seeded constructs supply living cells and could in principle introduce biological adaptability rather than purely material-controlled release, provided that the transplanted cells survive and remain functional after implantation.

The key distinction is therefore not simply weak versus strong attachment but whether release is governed by carrier properties set at fabrication—and modulated only incidentally by local conditions—or deliberately triggered by a defined tissue signal (Section 1.2.3). Increasing binding strength can reduce burst release and extend signal persistence, but it does not necessarily allow the system to determine whether a signal is needed at a particular stage of healing.

Passive biofunctionalization can therefore specify what signal is presented, approximately where it is presented, and for how long it is expected to remain available. What it generally cannot specify is when that signal should become active in response to the evolving biological state of the wound. This limitation provides the conceptual transition from conventional biofunctionalization to the bioresponsive polymer systems discussed later in this review.

## 5. Clinical Translation and the Substitute Gap

The clinical literature on oral soft tissue substitutes includes randomized trials, pairwise meta-analyses, non-inferiority designs, and network meta-analyses directly comparing substitute materials with autogenous grafts. Taken together, these studies show that polymer-based substitutes can provide clinically meaningful soft tissue augmentation while reducing donor-site morbidity, but they do not consistently reproduce the gains in mucosal thickness and keratinized tissue width achieved with autogenous tissue, whereas root-coverage outcomes depend on the material and protocol. Root coverage, keratinized tissue width, mucosal or gingival thickness, and soft tissue volume are distinct endpoints and are therefore reported separately below, and evidence from natural teeth and from implant sites is identified as such. Most direct comparisons of substitutes with connective tissue grafts for thickness and keratinized tissue width derive from peri-implant sites, whereas comparative evidence at natural teeth concerns predominantly root coverage.

### 5.1. Xenogeneic Collagen Matrices Versus Connective Tissue Grafts

A systematic review and meta-analysis of nineteen randomized and controlled clinical trials comparing collagen matrices with connective tissue grafts found greater mucosal thickness gain with connective tissue grafts by approximately 0.32 mm and greater keratinized tissue width gain by approximately 0.46 mm [102]. These pooled differences, derived from trials at both natural teeth and implant sites, indicate an advantage for autogenous tissue in both thickness and keratinized tissue width, although their clinical relevance should be interpreted in relation to the specific indication and baseline tissue dimensions. A separate meta-analysis of eight randomized trials restricted to peri-implant sites reported pooled three-month soft tissue thickness gains of 0.99 mm (95% CI, 0.78–1.20) with connective tissue grafts and 0.70 mm (95% CI, 0.47–0.93) with xenogeneic collagen matrices, a difference of 0.32 mm (95% CI, 0.17–0.47) favoring the graft, and concluded that xenogeneic matrices can increase soft tissue volume around implants while autogenous grafting remains superior [103].

Individual randomized trials show a similar direction of effect. In 60 patients undergoing soft tissue augmentation at implant uncovering, buccal soft tissue thickness increased by 1.2 ± 0.3 mm with connective tissue grafts and by 0.9 ± 0.2 mm with a xenogeneic collagen matrix at six months, corresponding to a 0.30 mm difference favoring the graft (95% CI, 0.2–0.5; *p* = 0.0001) [104]. In another parallel-group trial involving 30 patients treated at implant placement, thickness gains were 1.55 ± 0.11 mm with connective tissue grafts and 1.18 ± 0.11 mm with the collagen matrix, a difference of approximately 0.37 mm at three months (95% CI, 0.07–0.66; *p* = 0.016) [105].

A further systematic review similarly concluded that collagen matrices generally produce smaller increases in keratinized tissue width and soft tissue thickness or volume than autogenous grafts, while aesthetic outcomes may be comparable in selected indications [85]. A more recent systematic review and meta-analysis of peri-implant phenotype modification reached the same conclusion by endpoint, finding xenogeneic matrices less effective than connective tissue grafts for increasing buccal mucosal thickness and less effective than free gingival grafts for increasing keratinized mucosa width [106].

Thus, conventional xenogeneic collagen matrices can provide clinically useful augmentation, but direct comparative evidence, which at implant sites is the most abundant, generally favors connective tissue grafts when the primary endpoint is mucosal thickness or keratinized tissue width rather than root coverage.

### 5.2. Volume-Stable Collagen Matrices

Volume-stable collagen matrices were developed specifically to improve dimensional retention by prolonging scaffold persistence and increasing resistance to collapse. Their clinical performance is therefore particularly relevant to the question of whether improved material stability can reduce the difference from autogenous tissue.

In a multicenter, parallel-group randomized non-inferiority trial, peri-implant mucosal thickness was compared between a crosslinked volume-stable collagen matrix and connective tissue grafts. Ninety-eight patients completed this study, with 49 participants in each group. At 12 months, mucosal thickness increased by 1.0 ± 0.75 mm with connective tissue grafts and by 0.66 ± 0.58 mm with the matrix. The difference favoring the graft was approximately 0.37 mm (95% CI, 0.13–0.61; *p* = 0.002), and the predefined criterion for non-inferiority was not met [86].

This finding is important because increasing scaffold stability improved the material’s capacity for space maintenance but did not establish equivalence in peri-implant mucosal thickness with autogenous tissue. However, the result should not be interpreted as evidence that crosslinking or prolonged persistence is intrinsically ineffective. Rather, it indicates that improving volumetric stability may be insufficient to reproduce the combined biological properties of an autogenous graft. The evidence is not entirely uniform on this point: the same systematic review that found conventional matrices inferior for mucosal thickness reported no statistically significant difference between volume-stable matrices and connective tissue grafts for that endpoint [106], indicating that the magnitude of the difference may depend on the specific material and on the endpoint assessed.

### 5.3. Acellular Dermal Matrices

The evidence for acellular dermal matrices is less uniform. A meta-analysis evaluating immediate implant placement with simultaneous soft tissue augmentation found mean thickness gains of 0.46 ± 0.53 mm with connective tissue grafts and 0.33 ± 0.44 mm with acellular dermal matrices at 12 months [107]. The numerical difference of approximately 0.13 mm favored autogenous tissue but was not statistically significant, and substantial heterogeneity limited the certainty of the comparison.

This result is important because it demonstrates that the magnitude of the substitute–graft difference is not constant across material classes or clinical settings. Acellular dermal matrices may approach autogenous graft performance in selected indications, and their results should not be incorporated uncritically into a universal numerical “substitute gap”.

### 5.4. Network Evidence: Material, Endpoint, and Follow-Up

A network meta-analysis of 23 studies comprising 573 treated sites compared autogenous grafts with acellular dermal matrices, xenogeneic collagen matrices, and volume-stable collagen matrices [84]. Connective tissue grafts ranked highest for increasing peri-implant soft tissue thickness at both 180 and 360 days, whereas acellular dermal matrices performed favorably for buccal thickness gain, particularly during earlier follow-up. For keratinized mucosa augmentation, vestibuloplasty combined with free gingival grafting ranked highest at 180 days.

These findings reinforce two points. First, no single substitute performs equivalently across all dimensional endpoints. Second, the relative performance of materials may vary with follow-up time. The latter observation is particularly relevant to degradable scaffolds because early space maintenance does not necessarily predict the dimensions achieved after tissue remodeling.

However, network rankings should not be interpreted as demonstrating a fixed numerical difference between autogenous grafts and all substitutes. They provide comparative ordering across heterogeneous interventions rather than a common estimate of the magnitude of a “substitute gap”.

### 5.5. Autologous Platelet Concentrates as Biological Alternatives

Autologous platelet concentrates, particularly platelet-rich fibrin, differ conceptually from polymer matrices because their primary function is biological rather than structural. Randomized trials and meta-analyses indicate benefits in several early postoperative outcomes, including epithelialization, postoperative pain, edema, and patient-reported morbidity [108,109].

When platelet-rich fibrin has been compared with subepithelial connective tissue grafting for recession treatment, systematic evidence suggests similar performance for several root-coverage and periodontal outcomes but a less consistent effect on dimensional endpoints, particularly keratinized tissue width [110].

Individual randomized trials support this distinction. In a 12-month split-mouth study involving 59 teeth in 12 patients with multiple adjacent recessions, connective tissue grafting produced greater increases in gingival thickness and keratinized tissue width than leukocyte- and platelet-rich fibrin [111]. A separate split-mouth trial involving 72 recessions found lower residual recession depth and better attachment outcomes with connective tissue grafts at 16 months, together with greater early gingival thickness [112]. Similarly, a double-blind randomized trial comparing ozone-enriched advanced platelet-rich fibrin with grafting showed improvement in both groups but greater gains in gingival thickness and keratinized tissue width with the autogenous graft [113].

These studies suggest that an intervention may improve wound healing or recession coverage without necessarily reproducing the volumetric or dimensional effect of autogenous connective tissue. This distinction is central to soft tissue augmentation, where the intended outcome is often not simply wound closure or root coverage but the creation and maintenance of additional tissue volume.

### 5.6. The Substitute Gap by Endpoint and Site: What the Clinical Evidence Supports

**Figure 3:**
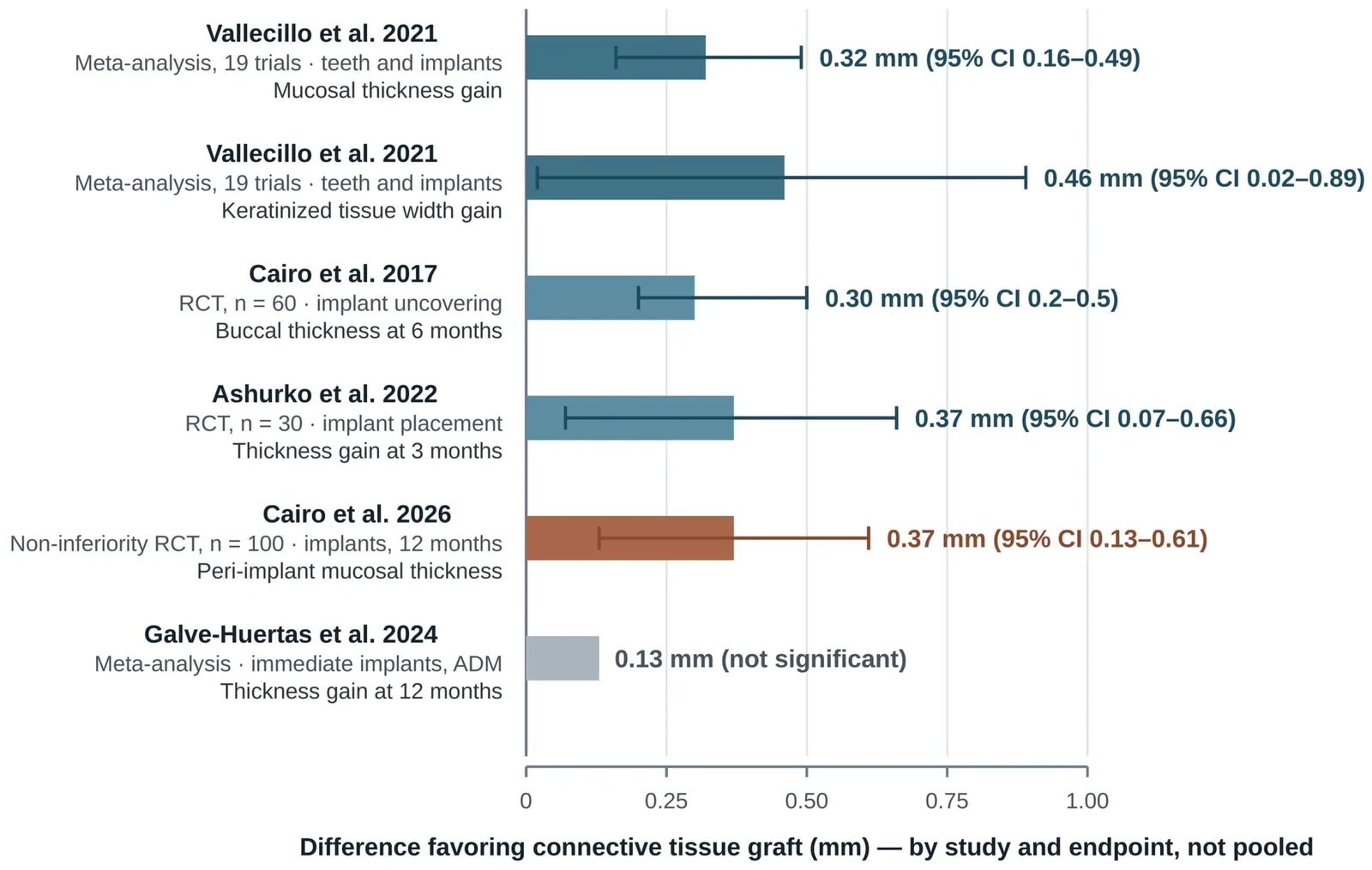
Selected differences in soft tissue thickness and keratinized tissue width gain between autogenous grafts and substitute materials. Estimates are presented separately by study and endpoint and do not represent a pooled analysis. Bars show the mean difference favoring the connective tissue graft; the whisker shows the 95% confidence interval where reported; the grey bar denotes a non-significant difference. Data are taken fron references [86,102,104,105,107] as summarized in Table 4. ADM, acellular dermal matrix; CI, confidence interval; CTG, connective tissue graft; RCT, randomized controlled trial.

The comparative evidence supports a consistent direction of effect more strongly than it supports a universal numerical difference. In several direct comparisons of xenogeneic or volume-stable collagen matrices with connective tissue grafts, the advantage of autogenous tissue in mucosal thickness gain is approximately 0.3–0.4 mm [85,86,102,103,104,105] (Table 4, Figure 3). However, this magnitude is not universal: the difference reported for acellular dermal matrices is smaller and non-significant in the available meta-analysis [107], while network meta-analysis provides relative rankings rather than a common effect estimate [84]. These comparisons concern peri-implant sites, with the exception of the pooled analysis by Vallecillo et al. [102], which included trials involving natural teeth and implants. The endpoints should not be conflated: the substitute gap is documented for mucosal thickness and keratinized tissue width, whereas root-coverage outcomes at natural teeth are material- and protocol-dependent. Selected substitutes or biofunctionalized constructs may approach the outcomes of connective tissue grafts, whereas others yield lower mean or complete root coverage (Section 5.5 and Section 6). Absence of a statistically significant difference should not be interpreted as equivalence unless equivalence or non-inferiority was formally tested, a caveat that applies particularly to small trials and to cell-seeded constructs. Long-term volumetric stability has been examined in only a few studies (Section 7.1).

This distinction is clinically important. Substitute materials reduce donor-site morbidity, shorten surgery, provide essentially unlimited availability, and may improve patient acceptance. Their value should not be judged exclusively by whether they reproduce every endpoint—thickness, keratinized tissue width, and volume—achieved with an autogenous graft. Nevertheless, where stable increases in thickness, keratinized tissue width, or soft tissue volume are the primary treatment objective, autogenous tissue remains the most predictable reference standard.

The biological and material basis of this difference cannot be established from comparative clinical studies alone. It would therefore be premature to attribute the substitute gap exclusively to scaffold degradation, lack of cellularity, insufficient growth-factor signaling, or absence of responsiveness. Several factors may contribute simultaneously, including scaffold architecture, mechanical stability, vascularization, cellular infiltration, degradation kinetics, wound contraction, and the biological activity of the carrier–host interface.

Autogenous connective tissue differs from an acellular substitute in several of these dimensions simultaneously: it contains living cells and native extracellular matrix, undergoes revascularization, integrates with the recipient site, and continues to remodel as part of the host tissue. A resorbable substitute, by contrast, must first be colonized and vascularized and must ultimately be replaced by newly formed tissue as its original structure is lost.

The clinically observed difference provides the problem that subsequent sections must explain, rather than proof of a single mechanism. The key question is whether further optimization of passive material properties and biofunctionalization can sufficiently narrow this gap, or whether additional benefit may require systems in which degradation, biological signaling, and cellular activity are more closely coupled to the evolving state of the healing tissue.

## 6. Biological Augmentation of Soft Tissue Matrices: Growth Factors, Autologous Concentrates, and Cells

Biological augmentation of oral soft tissue procedures has been investigated using autologous platelet concentrates, recombinant growth factors, bioactive protein preparations, and cell-based strategies. These approaches differ substantially in payload composition, dose control, persistence, and dependence on the carrier [114]. Their clinical evidence provides an opportunity to examine whether adding biological signaling can improve the performance of otherwise passive soft tissue matrices and, importantly, whether the effect depends on the interaction between the biological agent and the material used to deliver it.

### 6.1. Autologous Platelet Concentrates: Biological Complexity with Limited Temporal Control

Platelet-rich fibrin (PRF) is produced by centrifugation of whole blood without anticoagulant or exogenous thrombin, allowing formation of a fibrin network containing platelets, leukocytes, plasma proteins, and multiple signaling molecules. Its principal advantages are autologous origin, chairside preparation, low cost, and delivery of a physiologically complex mixture rather than a single recombinant factor.

Clinical evidence for PRF is strongest for early wound-healing and patient-centered outcomes, including epithelialization, postoperative pain, and edema [108,110]. A split-mouth randomized trial using three-dimensional facial scanning after mandibular third molar surgery similarly reported reduced postoperative swelling with platelet-rich fibrin, although not all comparisons reached statistical significance [115]. Its effect on tissue thickness and keratinized tissue width, the endpoints that define soft tissue augmentation, is less consistent.

The temporal behavior of conventional PRF may partly explain this distinction. Growth-factor release from the fibrin network is concentrated during the early healing period and may continue for approximately 10–14 days, while the fibrin scaffold itself undergoes progressive degradation over the following weeks [35]. This time window overlaps well with inflammation, angiogenesis, and early proliferative healing but only partially with the much longer remodeling phase that determines final tissue dimensions.

Comparative analyses of platelet concentrates prepared from the same donor blood have shown that different protocols yield different platelet, leukocyte, and growth-factor contents, with reduced-speed preparations containing comparable or higher levels of platelet-derived growth factors than platelet-rich plasma [116]. Several modifications of platelet concentrates have therefore sought to alter cellular content, fibrin architecture, injectability, or persistence (Table 5). Reduced centrifugal force can increase retention of platelets and leukocytes and generate a more porous fibrin architecture, whereas injectable formulations facilitate incorporation into matrices and irregular defects. These modifications alter biological composition and handling but do not necessarily provide prolonged structural persistence.

A different strategy is thermal modification of the platelet-poor plasma fraction. Heating induces albumin denaturation and formation of a more persistent gel that can subsequently be recombined with an unheated cell-rich fraction [117]. Reported formulations may remain structurally detectable for substantially longer periods than conventional fibrin preparations and have shown sustained release of TGF-β1 and PDGF together with effects on fibroblast proliferation and collagen expression [117].

This approach illustrates an important materials-design trade-off. Thermal stabilization increases persistence, but heating also reduces the biological activity of the treated plasma fraction [118]. Biological functionality must therefore be restored through combination with an unheated cellular or platelet-rich component. Structural persistence and biological activity are thus not automatically coupled simply by modifying the same material.

A further limitation of autologous platelet preparations is dose standardization. Centrifuge geometry and protocol have been shown to alter the cell content, growth-factor release, and fibrin architecture of a leukocyte- and platelet-rich fibrin clot, so that nominally identical preparations differ according to the device used [119]. Centrifugation protocol, device geometry, tube composition, timing, and individual hematological characteristics can influence the final preparation. Consequently, the concentration and effective dose of individual biologically active components are considerably less controlled than in recombinant-factor delivery.

Autologous concentrates therefore demonstrate that a resorbable biological carrier can improve selected aspects of healing. They do not, however, consistently demonstrate that early biological stimulation translates into greater long-term soft tissue thickness or keratinized tissue width.

**Table 5:** Selected platelet-rich fibrin formulations relevant to soft tissue biofunctionalization. Variants are distinguished by processing rather than chronology. Structural persistence and biological activity are reported separately, since they are not equivalent properties and are not extended together by any single modification.

Variant	Key Processing Feature	Physical Form	Approximate Structural Persistence	Biological Activity/Release Characteristics	Main Limitation	Ref.
L-PRF	Standard fixed-angle centrifugation	Solid membrane; dense fibrin network	~2–3 weeks	Growth factors release over approximately 10–14 days	Short structural persistence relative to remodeling	[108]
A-PRF/A-PRF+	Reduced relative centrifugal force	Solid; looser, more porous network	~2–3 weeks	Greater retained leukocyte and platelet fraction; enhanced cellular penetration	Persistence not extended by the modification	[116]
i-PRF	Short low-speed spin; liquid phase	Injectable liquid; gels in situ	Days	Suitable for matrix biofunctionalization; release largely early	Very short residence; dose unquantified	[35]
C-PRF	High-force fractionation of the buffy coat	Liquid; cell-concentrated	Days	Maximal cell and growth-factor yield per unit volume	Liquid form; no structural contribution	[120]
Alb-PRF	Platelet-poor plasma heated to 75 °C for 10 min, recombined with the buffy coat	Denatured albumin gel with cellular fraction	~4–6 months	Prolonged resorption; biological activity derives from the recombined unheated fraction, heating markedly reducing TGF-β activity of the plasma component	Structural persistence and biological activity arise from different fractions	[117,118]

### 6.2. Recombinant Human PDGF-BB

Platelet-derived growth factor-BB is chemotactic and mitogenic for fibroblastic cell populations relevant to periodontal wound healing and can also influence angiogenic processes. Its combination with collagen-based matrices provides one of the clearest clinical examples of recombinant growth-factor biofunctionalization in oral soft tissue augmentation.

In a triple-blind randomized placebo-controlled trial, 30 patients with multiple adjacent recession defects were treated with a coronally advanced flap and a crosslinked collagen matrix soaked either in rhPDGF-BB or saline [121]. At six months, mean root coverage was 88.25% in the rhPDGF-BB group compared with 77.72% in the control group. Gingival thickness gain was 0.80 mm and 0.51 mm, respectively (*p* = 0.01), and additional advantages were reported for volumetric and aesthetic outcomes as well as early postoperative recovery.

These findings demonstrate that biological functionalization can measurably improve the performance of a collagen matrix under specific experimental conditions. They do not, however, establish that rhPDGF-BB universally reproduces the biological effect of autogenous tissue or that the same benefit occurs with other carriers.

Systematic evidence places this result in a broader context. Reported mean root coverage was comparable among connective tissue grafting, rhPDGF-BB combined with connective tissue grafting, and rhPDGF-BB combined with a collagen matrix, whereas collagen matrix alone showed less recession reduction [122]. This suggests that rhPDGF-BB may improve the performance of an otherwise less effective scaffold, although the magnitude and persistence of that effect require consideration of the carrier and follow-up interval.

### 6.3. Long-Term Behavior: What Remained at Three Years?

Long-term follow-up of the same cohort is particularly informative because it separates an early biological effect from persistent dimensional superiority. Twenty-six of the original 30 participants were evaluated at three years [123]. Longitudinal analysis demonstrated favorable overall recession reduction, clinical attachment gain, mean root coverage, and complete root coverage in the rhPDGF-BB group across the observation period.

Both treatment arms remained stable over the three years: more than 90% of treated sites in both groups showed a stable gingival margin (change ≤ 0.5 mm), and mean root coverage decreased only minimally and non-significantly from six months to three years in either group (−7.5% and −6.7%). The changes from six months to three years did not differ between the groups for clinical, volumetric, aesthetic, or patient-reported outcomes. The advantage established at six months was, however, maintained rather than lost: across the complete observation period, the rhPDGF-BB group retained greater recession reduction, attachment gain, mean and complete root coverage, volume, and aesthetic scores, and mean root coverage at three years remained higher (81.0% versus 72.7%). Volumetric analysis showed small, non-significant reductions in both groups between six months and three years.

This result should be interpreted cautiously in both directions. It does not demonstrate loss of the tissue initially gained with rhPDGF-BB; nor, with four of 30 patients lost to follow-up and no connective tissue graft arm, does it establish that the growth factor altered the long-term trajectory of remodeling rather than improving the efficiency of the initial healing response. What it shows is that an early advantage obtained with a bioactive carrier persisted for three years once the tissue had matured, with parallel stability in both arms.

The finding nevertheless raises an important materials question: does extending or intensifying biological signaling during early healing alter the long-term trajectory of tissue remodeling, or primarily improve the efficiency of the initial healing response? Longer-term controlled studies are required to distinguish these possibilities.

### 6.4. The Agent - Carrier Pairing as Therapeutic Unit 

The most informative studies for understanding biological functionalization are those in which the carrier and surgical procedure are identical in both groups and the biological agent is the principal variable. Such designs make it possible to evaluate the added effect of biofunctionalization separately from the effect of the scaffold itself.

The rhPDGF-BB study described above provides a positive example: addition of the factor to a crosslinked collagen matrix improved several six-month clinical and volumetric outcomes [121].

Trials using different scaffolds have, however, reported different effects of rhPDGF-BB. These findings are consistent with carrier-dependent efficacy, but differences in patient populations, surgical protocols and study design prevent attribution of the contrasting outcomes to the carrier alone. In a split-mouth study of 40 bilateral recession defects in 17 patients, an acellular dermal matrix hydrated with rhPDGF-BB produced 69.0% root coverage compared with 76.7% for the same matrix hydrated with saline, with no significant benefit attributable to the growth factor [124].

This contrast is particularly important. A biological molecule cannot be considered independently of the material through which it is delivered. Carrier architecture, surface chemistry, binding affinity, porosity, hydration behavior, degradation, and volumetric stability may influence how much biologically active molecule remains locally available and how the surrounding cells respond to it. The bioactive agent and carrier therefore constitute a functional therapeutic pairing.

A similar carrier-dependent pattern is suggested by studies of platelet concentrates. Two randomized trials evaluating platelet-rich fibrin in combination with a volume-stable collagen matrix found no clear additional clinical benefit compared with the matrix alone. In a three-arm study of 75 patients, recession reduction at six months did not differ significantly between coronally advanced flap alone, flap with a volume-stable matrix, and flap with the matrix plus injectable PRF, and the authors concluded that adding i-PRF may not improve root coverage outcomes [125]. Gingival thickness gain, however, differed between groups, being greatest in the biofunctionalized arm and significantly greater than with the flap alone, although the matrix-only and biofunctionalized arms did not separate from one another. The addition therefore influenced a dimensional rather than a coverage endpoint and did so without clearly exceeding the contribution of the matrix itself. A split-mouth trial involving 24 patients likewise found no significant improvement in clinical, aesthetic, or patient-reported outcomes when the matrix was impregnated with liquid PRF [126].

By contrast, a randomized split-mouth study combining platelet-rich plasma with an acellular dermal matrix reported greater mean root coverage than the same matrix without plasma at 12 months [127]. Because platelet-rich plasma and platelet-rich fibrin differ in preparation and composition, and the carriers also differed between studies, these findings should not be interpreted as direct contradictions. They instead illustrate the difficulty of attributing an outcome to the biological adjunct without considering the complete formulation.

FGF-2 provides another promising but still limited body of evidence. A first-in-human, single-arm study of an rhFGF-2-impregnated collagen membrane in 21 patients (34 sites) reported reduced recession depth and increased keratinized tissue width at six months, but without a comparator, the contribution of the growth factor cannot be separated from that of the membrane or the flap [128]. A subsequent parallel-group randomized trial in 24 patients, one recession each, reported greater recession reduction, keratinized tissue gain, attachment gain, and phenotype modification with an FGF-2-functionalized collagen membrane than with the plain membrane [129]. These studies support biological activity but remain small and short-term, and the specific carrier systems differ from contemporary volume-stable matrices.

Cell seeding represents an additional type of carrier–biology interaction. In a split-mouth randomized study, a volume-stable collagen matrix seeded with autologous gingival fibroblasts produced greater clinical attachment gain and greater reductions in recession depth and width than the identical unseeded scaffold [130]. A possible explanation is that the biological component is no longer a finite molecular payload but a living cell population capable of producing extracellular matrix and responding to local signals. However, the trial did not examine whether the seeded cells survived or remained active after implantation, so the clinical difference cannot be attributed to adaptive cellular signaling. The clinical evidence remains preliminary, but the approach provides proof of concept that modification of the cellular content of a scaffold can influence its clinical performance.

Other combinations currently have less robust evidence. A prospective case series using a volume-stable matrix soaked in a polynucleotide–hyaluronic acid formulation reported favorable root coverage and gingival thickness at 12 months [131]; however, without a matrix-only comparator, the contribution of the biological formulation cannot be separated from that of the surgical procedure and scaffold.

Taken together, the available studies support a hierarchy of evidential confidence rather than a hierarchy of biological potency. rhPDGF-BB delivered from a crosslinked collagen matrix has controlled evidence of benefit; FGF-2 and fibroblast-seeded matrices show promising clinical signals from single small trials; other formulations are supported by smaller, single, or uncontrolled studies; and several PRF-matrix combinations have not demonstrated additional benefit over the carrier alone.

### 6.5. Enamel Matrix Derivative as a Bioactive Adjunct

Enamel matrix derivative (EMD) is not a growth factor but a complex biological preparation and is included here because it provides another clinically relevant example of adjunctive biological signaling in periodontal plastic surgery.

Meta-analytic evidence indicates that EMD can improve selected outcomes when added to a coronally advanced flap, whereas its additional benefit becomes less evident when the procedure already includes an autogenous connective tissue graft [132]. Individual randomized studies have produced variable results, with some reporting improved root coverage and others finding limited or no additional benefit over connective tissue grafting alone [133,134,135].

The same uncertainty is evident when EMD is combined with substitute matrices. In a four-arm randomized study comparing coronally advanced flap alone, flap with a collagen matrix, flap with EMD, and flap with both components, the combination did not clearly outperform either bioactive adjunct individually [136]. A meta-analysis of four randomized trials evaluating EMD combined with acellular dermal matrices found no statistically significant improvement in root coverage or other major periodontal outcomes [137]. Similarly, coating a xenogeneic dermal matrix with enamel matrix proteins did not produce a significant clinical advantage over the uncoated scaffold at one year [138].

The EMD literature therefore reinforces a broader observation: adding biological signaling does not necessarily produce an additive clinical effect when the underlying surgical procedure or carrier already provides substantial regenerative support.

### 6.6. Why Have Biological Adjuncts Not Replaced Autogenous Grafting?

The available evidence suggests several reasons why growth factors and other biological adjuncts have not displaced autogenous connective tissue grafting in soft tissue augmentation.

First, biological effects are often most evident during early or intermediate healing. Improvements in wound healing, postoperative morbidity, or six-month clinical outcomes are relevant, but the thickness, width, and volume objectives of soft tissue augmentation require stability during subsequent remodeling and long-term maintenance [139,140]. Evidence beyond the first year remains comparatively limited for most biofunctionalized matrices.

Second, biological efficacy is carrier-dependent. The contrasting results obtained with rhPDGF-BB on different scaffolds, together with variable findings for platelet preparations, indicate that the carrier is not a neutral vehicle (Table 6). Material composition, architecture, degradation, and molecular retention influence the biological intervention itself.

Third, adding a biological signal does not reproduce the complete biological constitution of an autogenous graft. A connective tissue graft provides native extracellular matrix, resident cells, vascular structures capable of revascularization, and subsequent remodeling within the host [141]. A recombinant factor can modulate selected cellular processes, but it does not independently provide cellularity, vascular architecture, mechanical persistence, or tissue organization.

Fourth, the clinical benchmark is demanding. Autogenous connective tissue grafting is established, comparatively inexpensive, and supported by long-term clinical evidence [72]. A biologically functionalized substitute must therefore provide a meaningful improvement in thickness, keratinized tissue width, volume stability, morbidity, predictability, or workflow to justify additional manufacturing complexity and cost.

These observations indicate that biological augmentation should be considered within the context of the complete material system and the time-dependent requirements of healing. This shifts the design problem from identifying an increasingly potent molecule toward engineering a material–biology system whose components function coherently throughout healing and remodeling.

### 6.7. Quality and Independence of the Comparative Evidence

The comparative evidence summarized in Table 4 and Table 6 should be read with its limitations in view. The individual trials are small: the largest direct comparison of a xenogeneic collagen matrix with a connective tissue graft at implant sites randomized 60 patients [104], the volume-stable matrix trial analyzed 98 patients who completed 12 months [86], and the trial at implant placement included 30 patients followed for three months [105]. Follow-up is short relative to the remodeling period over which augmented volume is lost: three to six months in most trials, twelve months in the non-inferiority trial [86], and three years in only one of the biofunctionalization trials in Table 6 [123]. Thickness was measured by different methods—transmucosal probing with a needle and caliper [86,104], superimposition of scanned models on cone-beam tomography [105], intraoral scanning and ultrasonography [121]—which are not interchangeable and have different reproducibility, so that between-group differences of 0.3–0.4 mm lie close to the resolution of the needle method. Attrition was modest in the trials for which this information was accessible to us (two of sixty patients in the xenogeneic matrix trial [104]; two of hundred in the non-inferiority trial [86]; none in the trial at implant placement [105], the fibroblast-seeded matrix trial [130] or the four-arm enamel matrix derivative trial [136]; one of twenty-five in the split-mouth PRF trial [126]; none at six months and four of thirty at three years in the rhPDGF-BB trial [121,123]), but was not reported in the abstracts of several others, and for these the handling of missing data could not be assessed.

The unit of analysis deserves particular attention. In the peri-implant trials in Table 4, one implant per patient was treated, and the patient was the statistical unit [86,104], so that sites and patients coincide. In several of the recession trials in Table 6 and in the cell-seeding trials of Section 8.5, by contrast, multiple adjacent recessions or bilateral split-mouth sites were treated in the same patient, whereas three trials treated one recession per patient [125,129,136]. Two trials explicitly modeled the correlation between sites within a patient: the rhPDGF-BB trial, using linear mixed-effects models for 91 sites in 30 patients [121,123], and the acellular dermal matrix trial, with teeth nested within patients [124]. The split-mouth PRF trial [126] and the most recent cell-seeding trial [130] entered one site per side into a paired analysis. By contrast, the two earlier cell-seeding trials (Section 8.5) compared the sides with unpaired tests (Mann–Whitney or Student’s *t*-test) despite the split-mouth design; the platelet-rich plasma trial analyzed 84 recessions in 12 patients as independent observations [127], and the meta-analysis of enamel matrix derivative pooled 194 defects from 58 patients across four split-mouth trials without describing a paired analysis [137]. Where sites within a patient are treated as independent observations, confidence intervals are too narrow and *p*-values too small [142,143], so the statistical significance of small differences in these studies should be interpreted with caution.

Finally, the systematic reviews cited in this section draw largely on the same primary trials and should not be counted as independent confirmations of one another. The trial of Cairo et al. [104] is included in each of the pairwise meta-analyses of collagen matrices cited here [85,102,103,106] and in the network meta-analysis [84]. The Zurich trial of a volume-stable collagen matrix in 20 patients is represented by several reports at different follow-up intervals (Section 7.1); Vallecillo et al. counted four of these reports as separate studies among their nineteen [102], and two other reviews counted two [103,106]; counting several reports of one trial as independent studies inflates the weight of that trial’s data in a pooled estimate [144]. The peri-implant thickness meta-analyses of Ríos-Osorio et al. [106] and Ashurko et al. [103] share seven of the eight trials in the latter. The near-identical pooled differences of approximately 0.3 mm reported by these reviews [102,103,106] therefore reflect the same data analyzed repeatedly rather than replication across independent trial sets. The meta-analysis of acellular dermal matrices at immediate implants [107] is the exception, being based on trials that do not overlap with the collagen-matrix reviews. Agreement among reviews is accordingly weaker evidence than it appears, and the consistent direction of effect summarized in Section 5.6 rests on a small number of small, short trials.

## 7. The Biological Limitations of Passive Soft Tissue Substitutes

The preceding sections considered soft tissue substitutes from three complementary perspectives: the material platforms available, the strategies used to biofunctionalize them, and their comparative clinical performance. Taken together, these data identify several recurring limitations that may constrain the long-term performance of conventional passive scaffolds. These limitations are interrelated and arise from the challenge of coordinating scaffold persistence, tissue formation, and biological signaling within a healing environment that changes over time.

### 7.1. Volumetric Instability

A central challenge in soft tissue augmentation is the maintenance of the volume initially created by the procedure. Clinical volumetric studies have shown that substantial changes in contour and tissue thickness can occur during the first months after augmentation, with the magnitude of reduction differing between autogenous grafts and collagen-based substitutes [139,140].

This early dimensional change reflects several processes occurring simultaneously, including scaffold hydration and compression, enzymatic degradation, cellular infiltration, extracellular matrix formation, vascular remodeling, and wound contraction. The relative contribution of each mechanism is difficult to isolate clinically, but the overall result is that initial scaffold volume does not translate directly into equivalent long-term tissue volume. The challenge is to maintain the space until sufficient host tissue has formed and matured to support it independently.

### 7.2. Degradation Is Not Necessarily Coupled to Tissue Maturation

Resorbable matrices are progressively degraded through enzymatic, hydrolytic, or combined mechanisms depending on their composition. In collagen-based scaffolds, degradation is strongly influenced by the local proteolytic environment, whereas synthetic biodegradable polymers may follow predominantly hydrolytic pathways [76,80,145].

Although degradation is influenced by the host environment, conventional scaffold resorption is not deliberately coupled to the degree of cellular colonization, vascularization, or extracellular matrix maturation. Preclinical observations indicate that material loss and tissue ingrowth proceed concurrently, but not necessarily at matched rates [75,146].

If degradation proceeds faster than tissue replacement, dimensional loss may occur. Conversely, excessive persistence may interfere with integration or maintain a foreign-body interface longer than required. Crosslinking can shift this balance by increasing resistance to enzymatic degradation, but its effects depend on crosslinking chemistry and density and may involve trade-offs between persistence, cellular infiltration, vascularization, and host response.

### 7.3. Limited Capacity for Feedback

Conventional passive scaffolds are designed with predefined material properties. They may be influenced by the biological environment, but they do not contain an engineered mechanism for detecting the biological state of the wound and modifying their behavior accordingly.

For example, a conventional scaffold does not actively determine whether inflammation remains elevated, vascularization is insufficient, proteolytic activity has increased, bacterial challenge is present, or tissue maturation is delayed. It cannot deliberately adjust growth-factor release, crosslink cleavage, swelling, antimicrobial activity, or mechanical behavior in response to such changes.

As defined in Section 1.2.3, host-mediated degradation alone does not make a material responsive: a material may be biologically influenced without possessing a designed trigger and response.

### 7.4. Dependence on Host Cellular Colonization

Autogenous connective tissue grafts and acellular substitutes differ fundamentally in their cellular composition. An autogenous graft enters the wound with native extracellular matrix and resident cells, whereas an acellular matrix depends on recruitment of host fibroblasts, endothelial cells, and other populations from the recipient site [5,141]. The success of an acellular scaffold depends on the rate and extent of host–cell migration and vascular penetration relative to scaffold degradation. In thicker or poorly vascularized regions, insufficient cellular penetration may limit complete replacement of the implanted material by organized connective tissue.

This does not mean that acellular matrices necessarily fail to integrate; many support substantial host colonization. However, cell recruitment represents an additional biological requirement that is largely absent from an autogenous graft at the time of implantation. Cell-seeding strategies directly address this limitation by introducing fibroblasts, stromal cells, endothelial cells, or combinations thereof before implantation. Their potential and limitations are discussed further in Section 8.

### 7.5. A Single Predefined Architecture for a Multiphase Healing Process

Soft tissue healing is temporally heterogeneous. Early phases require clot stabilization, inflammatory resolution, vascular penetration, and rapid cellular migration, whereas later phases require mechanical stability, extracellular matrix organization, and controlled remodeling.

A homogeneous scaffold with fixed architecture may not be optimal for all of these requirements simultaneously. High porosity may facilitate cellular and vascular infiltration but reduce mechanical stability; increased density or crosslinking may improve space maintenance but impede biological penetration [76,147]. Layered, gradient, and spatially patterned constructs can partly address this conflict by assigning different functions to different regions of the scaffold.

### 7.6. The Long-Term Maintenance Challenge

Peri-implant soft tissue introduces an additional requirement because the transmucosal interface must remain functional long after the initial wound has healed. The tissue must maintain epithelial attachment, connective tissue organization, and resistance to continuous microbial and mechanical challenge around a permanently exposed biomaterial surface [3,5].

A temporary augmentation scaffold can influence initial tissue formation, but its direct biological contribution progressively diminishes as the material resorbs. Long-term peri-implant stability depends ultimately on the quality and organization of the host tissue that replaces or integrates with the scaffold. This creates a distinct design challenge: materials must support not only short-term healing but the formation of tissue capable of maintaining a stable transmucosal interface after the material itself is no longer present.

### 7.7. Sourcing, Batch Variability, and Reproducibility

Biological materials also introduce variability that is less prominent in fully synthetic systems. Xenogeneic and allogeneic scaffolds may differ according to tissue source, processing, decellularization, crosslinking, sterilization, and manufacturing batch [148,149]. Autologous platelet concentrates add variability related to preparation protocol and individual patient characteristics.

Such variability can influence mechanical properties, degradation, biological activity, and release behavior but is not always characterized at the point of clinical use. This complicates both comparison between studies and prediction of individual clinical performance.

Synthetic polymers offer greater control over composition and manufacturing parameters, but their reproducibility does not in itself overcome the biological limitations of behavior that is not coupled to the state of the wound.

### 7.8. A Translational Mismatch Between Scaffold and Signaling Research

The soft tissue augmentation literature has historically focused primarily on scaffold architecture, handling, dimensional outcomes, and comparison with autogenous grafts. In contrast, research on controlled biological signaling, responsive delivery, and adaptive polymer systems remains predominantly preclinical.

This creates a translational mismatch between two relatively mature research domains: clinically tested soft tissue matrices and increasingly sophisticated biomaterial strategies for controlled or stimulus-dependent signaling.

Growth-factor functionalization, platelet-concentrate loading, cell seeding, and responsive polymer technologies represent attempts to connect these fields, but the strength of clinical evidence varies substantially among them.

### 7.9. A Common Design Limitation Rather than a Single Failure Mechanism

The limitations described above should not be interpreted as eight independent failures, nor as evidence that all passive matrices fail through a single biological mechanism. They instead converge on a common design challenge: conventional scaffolds possess properties that are largely defined before implantation, whereas the biological environment they enter changes continuously during healing and remodeling.

Volumetric loss may reflect an unfavorable relationship between degradation and tissue replacement. Limited cellularity increases dependence on host colonization. Fixed architecture may not optimally support both early infiltration and later mechanical stability. Release kinetics set by the carrier may not correspond to changing biological requirements. These processes interact, and their relative importance is likely to vary among materials, indications, and patients.

The distinction is therefore not between a “functional” graft and a “non-functional” substitute but between a living tissue capable of continuous biological adaptation and an engineered scaffold whose principal properties are set by design and are not coupled to the state of the wound by any deliberate mechanism. This observation suggests two complementary directions for future material development. The first is to improve conventional scaffold design by optimizing architecture, degradation, mechanical properties, spatial organization, and biological functionalization. The second is to allow selected material functions—degradation rate, swelling, or release—to change in response to defined biological signals.

Cell-seeded constructs provide an intermediate concept, as they introduce living cells that, if they survive and remain functional after implantation, can respond to oxygen tension, mechanical strain, matrix stiffness, inflammatory mediators, and other environmental cues. Whether such biological responsiveness, or alternatively engineered stimulus-responsive polymers, can translate into more stable clinical soft tissue augmentation remains an open question. The engineering strategies developed to address this question are considered in the following section.

## 8. Beyond Passive Design: Engineering Strategies and Bioresponsive Systems

The limitations discussed in Section 7 can be addressed at two fundamentally different levels. The first is to improve a material without introducing an engineered response mechanism, for example by increasing persistence, introducing spatial organization, adding biological signals, or supplying cells. The second is to engineer the material itself so that selected functions change in response to conditions encountered after implantation.

These approaches should not be treated as equivalent: a more durable, more bioactive, or more complex scaffold is not necessarily a bioresponsive scaffold (Section 1.2.3).

### 8.1. Strategies Without an Engineered Response Mechanism

Several strategies can improve conventional soft tissue substitutes without introducing true stimulus-dependent behavior. Crosslinking and volume stabilization increase resistance to degradation and improve mechanical persistence. As discussed in Section 3 and Section 5, these modifications can improve space maintenance, but prolonged persistence alone has not consistently produced thickness gains equivalent to autogenous connective tissue. Crosslinking modifies the degradation trajectory; it does not inherently couple degradation to the biological progress of tissue replacement.

Layered and spatially organized scaffolds address the heterogeneity of the soft tissue interface by assigning different functions to different regions of the construct [150]. A superficial compartment may be optimized for epithelial interaction while a deeper region supports fibroblast migration and vascular penetration. Such spatial programming can reproduce biological heterogeneity more closely than a homogeneous scaffold, but the architecture is not designed to change in response to the tissue.

Growth-factor and peptide functionalization can enhance cellular adhesion, migration, angiogenesis, or matrix formation. Affinity-based delivery and covalent immobilization provide greater control over signal retention and spatial presentation than simple adsorption. Nevertheless, the timing and magnitude of biological activity remain predominantly determined by the original material design. Autologous platelet concentrates provide a clinically accessible multifactorial biological stimulus and can modify early healing and angiogenic behavior [151]. Their limitations include biological variability, incomplete dose standardization, and predominantly early activity.

Gene-, RNA-, and extracellular vesicle-based approaches represent additional strategies for extending or modifying endogenous signaling [152]. Mesenchymal stromal cell-derived extracellular vesicles, for example, have shown pro-angiogenic and immunomodulatory effects in periodontal models [153]. These approaches may prolong or diversify biological signaling but should still be distinguished from materials whose output is directly triggered by the current state of the surrounding tissue.

Collectively, these strategies demonstrate the substantial design space available to passive materials. They can modify what a scaffold contains, how long it persists, where a biological signal is located, and how that signal is released, but not whether it is released in response to the state of the wound.

### 8.2. Bioresponsive Polymer Systems: Degradation and Release Triggered by the Wound Environment

A bioresponsive polymer is designed to detect a change in its environment and translate that change into a physicochemical response that alters material behavior or therapeutic output [154,155]. The basic sequence can be described as stimulus recognition → transduction → response.

Potential responses include cleavage of crosslinks, changes in swelling or charge, altered permeability, accelerated or reduced degradation, exposure of previously hidden ligands, or release of a therapeutic payload. All biodegradable materials interact with their surroundings. However, responsiveness requires that a defined environmental condition be intentionally incorporated into the design as a trigger for a functional material response. Thus, enzymatic degradation of an ordinary collagen scaffold by host collagenases represents host-dependent degradation, whereas a polymer containing a deliberately engineered enzyme-cleavable linker uses enzymatic activity as a designed input signal.

For oral soft tissue augmentation, such systems are conceptually attractive because the wound and peri-implant environments provide several potential biological inputs, including changes in proteolytic activity, reactive oxygen species, microbial virulence factors, oxygen tension, local chemical conditions, and mechanical loading. The principal engineering challenge is specificity: a clinically useful system must discriminate biologically meaningful changes from the considerable physiological variability of the oral environment.

### 8.3. Biochemical, Enzymatic, and Microbial Triggers

#### 8.3.1. pH-Responsive Systems

pH-responsive polymers change ionization, swelling, solubility, or molecular interactions according to local acidity or alkalinity. pH-sensitive coatings on implant surfaces have been developed to combine targeted infection control with a fibroblast-compatible surface intended to support soft tissue integration [156]; the outcomes reported are antibacterial, in vitro cytocompatibility, and histological responses in a rat subcutaneous model, and no mucosal dimension was measured (Table 7). In the oral environment, their potential value lies in responding to disease-associated or biofilm-associated pH changes rather than to a universally acidic pathological state. A related layer-by-layer coating of poly(acrylic acid) and poly(L-lysine) carrying tetracycline complexed with anionic β-cyclodextrin has been reported to sustain antibacterial release for approximately 30 days while remaining compatible with human gingival fibroblasts [157].

However, oral pH varies physiologically with diet, saliva, biofilm composition, and anatomical location. A pH-responsive oral system would therefore require carefully defined activation thresholds and characterization under repeated physiological pH cycling to minimize unintended release.

#### 8.3.2. Reactive Oxygen Species-Responsive Systems

Reactive oxygen species (ROS) increase during inflammatory responses and can therefore serve as endogenous biochemical triggers. Thioketal, boronate ester, and phenylboronic acid-related chemistries have been investigated as ROS-sensitive components of responsive materials [158,159]. Such systems can potentially combine two functions: consumption or neutralization of excess ROS and stimulus-dependent release of an anti-inflammatory, regenerative, or antimicrobial payload. This dual functionality is particularly relevant to chronically inflamed periodontal or peri-implant environments. The limitation is specificity. ROS participate in normal host defense and signaling as well as pathological inflammation, and responsive thresholds must therefore distinguish physiological oxidative activity from sustained pathological elevation [160].

#### 8.3.3. Enzyme- and MMP-Responsive Systems

Enzyme-responsive polymers are particularly relevant to tissue remodeling because proteolytic activity changes markedly during inflammation and extracellular matrix turnover. MMP-cleavable peptides can be incorporated as crosslinks so that elevated enzymatic activity modifies hydrogel structure, degradation, or cargo release. A collagenase-responsive hydrogel described for the treatment of periodontitis illustrates this principle: degradation is driven by the elevated proteolytic activity of the diseased site and releases nanoparticle-encapsulated inhibitor of inflammation-induced PERK signaling in periodontal ligament stem cells, with anti-inflammatory effects and reduced alveolar bone loss demonstrated in a rat periodontitis model; no soft tissue dimensional outcome was measured [26] (Table 7). For soft tissue augmentation, the broader design opportunity is to couple scaffold behavior to proteolytic conditions associated with tissue remodeling rather than leaving degradation governed only by the carrier’s intrinsic susceptibility to hydrolysis and host proteolysis.

#### 8.3.4. Systems Responsive to Pathogens and Their Virulence Systems

Microbial enzymes offer potentially greater biological specificity than generalized inflammatory signals. A material can, in principle, contain linkers cleaved preferentially by proteases associated with a particular pathogen, thereby releasing an antimicrobial agent only when the relevant microbial signal is present.

Gingipain-responsive hydrogel systems provide an example of this approach. A thermosensitive hydrogel containing a gingipain-cleavable sequence has been used to trigger release of the chemokine SDF-1 in the presence of Porphyromonas gingivalis, whose gingipains are major virulence factors [161], with in situ periodontal regeneration, assessed as alveolar bone and periodontal ligament outcomes, demonstrated experimentally; soft tissue dimensions were not assessed [27] (Table 7).

Pathogen-responsive systems are conceptually attractive for the transmucosal interface because they link therapeutic output to microbial challenge rather than to elapsed time. Their major translational challenge is that periodontal and peri-implant diseases are polymicrobial. Responsiveness to a single organism or virulence factor may therefore be insufficient to capture the biological complexity of clinical dysbiosis.

#### 8.3.5. Hypoxia-Responsive Systems

Hypoxia accompanies early wound healing and can become particularly relevant when cellular infiltration outpaces vascular ingrowth. Azobenzene-, nitroimidazole-, and related oxygen-sensitive chemistries have been investigated in responsive delivery systems [162]. A potential application in soft tissue augmentation would be to couple angiogenic signaling to insufficient oxygenation within the scaffold, thereby linking release to one of the conditions that may limit cellular survival and tissue replacement. At present, this remains primarily a conceptual extrapolation from responsive biomaterial systems developed in other tissues rather than an established strategy for oral soft tissue augmentation.

### 8.4. Mechanical, Multi-Stimulus, and Spatially Programmed Systems

Mechanical forces are integral to soft tissue healing. Cells sense matrix stiffness, strain, compression, and tension, and mechanotransduction influences fibroblast behavior, myofibroblast differentiation, extracellular matrix synthesis, and activation of latent signaling molecules [163].

Mechanoresponsive materials attempt to convert these physical inputs into a material response. Piezoelectric and mechanically activated systems can transform deformation into electrical, chemical, or release-related outputs [164]. Such concepts are relevant to oral soft tissues because an augmentation material experiences flap tension, wound contraction, mastication, and long-term functional loading. However, the specific use of mechanoresponsive polymers to control soft tissue volume or transmucosal integration remains largely unexplored, and mechanistic plausibility should not be presented as evidence of clinical efficacy.

Single-trigger systems also face a specificity problem. pH, ROS, enzymatic activity, and mechanical loading all fluctuate physiologically. Multi-stimulus systems can increase selectivity by requiring two or more conditions to coincide before a response is initiated [165]. This introduces a rudimentary form of biological logic and may reduce false activation, but it also increases fabrication complexity, characterization burden, and regulatory uncertainty.

A further step is feedback-controlled or self-regulating behavior, in which activation modifies the condition that initiated the response and the output subsequently decreases as the environment returns toward a target state [17,20]. Such systems should be distinguished from conventional responsive materials: a material that reacts to a stimulus is not necessarily self-regulating.

Spatial programming provides another dimension of control. Gradient and multilayer constructs can place different mechanical properties, ligands, cell-binding motifs, or release systems within different regions of the same scaffold [150]. This is particularly relevant to the transmucosal interface, where epithelial attachment and connective tissue organization represent biologically distinct zones.

Four-dimensional fabrication extends this concept by producing constructs that change shape or organization after placement in response to a predefined stimulus [166]. Such approaches offer sophisticated spatial and temporal control but remain substantially removed from routine clinical soft tissue augmentation.

Table 7 summarizes the representative systems cited in Section 8.2, Section 8.3 and Section 8.4, listing for each the material, the engineered trigger, the experimental model, the comparator, and the main reported outcome. Two features of this evidence should be made explicit. First, every primary study was performed in periodontitis or infection models or, for the implant coating, in a subcutaneous biocompatibility model, and the outcomes reported are antibacterial efficacy, reduced inflammatory infiltrate, osteogenic differentiation of periodontal ligament cells, or reduced alveolar bone loss. Second, none of these studies measured mucosal thickness, keratinized tissue width, or soft tissue volume stability. Reduced inflammation or periodontal regeneration in such models is not evidence of soft tissue augmentation: the clinical deficit that a substitute must overcome is a dimensional one (Section 5.6), and an anti-inflammatory or regenerative effect on bone or periodontal ligament does not imply that the material would increase or maintain the thickness, width, or volume of the mucosa. The systems in Table 7 therefore demonstrate that the stimulus–response principle can be implemented with triggers present in the oral environment, not that it improves the endpoints by which soft tissue augmentation is judged.

**Table 7:** Representative bioresponsive systems cited in Section 8.2, Section 8.3 and Section 8.4, with the trigger, model, comparator, and main reported outcome. One non-responsive controlled-release coating [157] is included for comparison. The final column records whether a soft tissue dimensional outcome (mucosal thickness, keratinized tissue width, or volume stability) was measured. For the reviewed classes without a primary study in oral soft tissue, the entry reflects the scope of the cited review. CEJ, cemento-enamel junction; MMP, matrix metalloproteinase; PDL, periodontal ligament; PEG, poly(ethylene glycol); ROS, reactive oxygen species; SDF-1, stromal cell-derived factor-1.

System	Engineered Trigger	Model	Comparator	Main Reported Outcome	Soft tissue Dimensional Outcome	Ref.
MMP-cleavable acrylated hyaluronic acid hydrogel loaded with nanoparticles of a PERK inhibitor	Elevated collagenase/MMP-2 activity cleaving peptide crosslinks	Human PDL stem cells stimulated with lipopolysaccharide in vitro; lipopolysaccharide-induced periodontitis in rats (6 per group, 21 days)	Sham, lipopolysaccharide alone, blank hydrogel, free drug (in vitro also nanoparticles without hydrogel)	Cumulative release 71% with versus 42% without collagenase at 21 days; restored osteogenic markers in vitro; reduced CEJ–alveolar crest distance and inflammatory infiltrate in vivo	Not measured (gingival swelling noted qualitatively only)	[26]
Gingipain-cleavable thermosensitive PEG hydrogel releasing an antimicrobial peptide and SDF-1	Arginine-specific gingipain of Porphyromonas gingivalis cleaving a peptide module	PDL stem cells and P. gingivalis in vitro; ligature- and P. gingivalis-induced periodontitis in Wistar rats (72 rats, 6 groups of 12), 1 and 4 weeks after injection	Healthy rats; untreated periodontitis; hydrogels without the cleavable peptide and/or without SDF-1	Release increased with gingipain concentration; inhibition of P. gingivalis; enhanced PDL cell proliferation, migration and osteogenic differentiation; smallest CEJ–alveolar crest distance and least inflammatory infiltrate with the complete hydrogel at 4 weeks	Not measured	[27]
pH-responsive poly(methacrylic acid) film over a layer-by-layer tetracycline–β-cyclodextrin coating on titanium	Acidic pH (release accelerated at pH 4.5 versus 7.4), as in infected or inflamed peri-implant sites	Saliva-derived peri-implantitis biofilms, human keratinocytes and gingival fibroblasts (monolayer and collagen matrix) in vitro; rat subcutaneous implantation, 7 animals per group, 2 and 7 days	Uncoated titanium; coating without the drug	Nearly 4-log reduction in biofilm colony counts versus uncoated titanium; no cytotoxicity up to 6 days; fewer inflammatory cells, more collagen and more CD206-positive macrophages at 7 days	Not measured (subcutaneous model without mucosa)	[156]
Layer-by-layer poly(acrylic acid)/poly(L-lysine) coating with tetracycline–β-cyclodextrin complexes (non-responsive comparator)	None; passive sustained release	Staphylococcus aureus and human gingival fibroblasts in vitro	Coatings without the drug complex	Antibacterial activity sustained to approximately 30 days; non-cytotoxic to gingival fibroblasts	Not measured	[157]
ROS-responsive thioketal-, boronate- and phenylboronic-acid-based carriers (reviewed class)	Elevated reactive oxygen species	Inflammatory and periodontal models reviewed in the cited sources; no primary study in oral soft tissue augmentation identified by our search	Varies by study	ROS scavenging and ROS-triggered release of anti-inflammatory or antimicrobial payloads	None reported in the reviewed studies	[158,159,160]
Hypoxia-responsive azobenzene- and nitroimidazole-based carriers (reviewed class)	Low oxygen tension	Non-oral tissue models reviewed in the cited source; no primary study in oral soft tissue augmentation identified by our search	Varies by study	Hypoxia-triggered release of angiogenic or therapeutic payloads	None reported	[162]
Mechano-, multi-stimulus and four-dimensional systems (reviewed classes)	Mechanical deformation; two or more coincident stimuli; predefined post-placement stimulus	Conceptual and non-oral models reviewed in the cited sources; no primary study in oral soft tissue augmentation identified by our search	Varies by study	Deformation converted into electrical, chemical or release outputs; reduced false activation; shape change after placement	None reported	[164,165,166]

### 8.5. Cell Seeding: Biological Responsiveness Without Engineered Sensing Chemistry

Cell seeding occupies a distinctive position within this framework. Rather than engineering a synthetic sensor into the polymer, the scaffold is populated with living cells that already possess endogenous sensing and response mechanisms.

Gingival fibroblasts respond to matrix stiffness, mechanical strain, oxygen tension, soluble mediators, and extracellular matrix composition and modify their proliferation, migration, secretory activity, and matrix production accordingly [163,167,168]. In culture, these cells can therefore confer biological adaptability on an otherwise passive scaffold without requiring the polymer itself to perform molecular sensing; whether seeded cells retain this capacity after implantation in the oral environment has not been directly demonstrated. Importantly, unlike engineered stimulus-responsive polymer systems, cell-seeded constructs have already entered clinical investigation in oral soft tissue applications.

A split-mouth randomized controlled pilot study in 11 patients (22 sites) using a collagen membrane seeded with autologous gingival fibroblasts under a coronally advanced flap reported greater percentage root coverage than the same membrane without cells, with no difference in gingival thickness [169]. A split-mouth randomized comparison in 18 patients of autologous fibroblast culture on a collagen scaffold with connective tissue grafting found both approaches clinically effective, although keratinized tissue width remained greater with the autogenous graft [170]. More recently, a split-mouth randomized trial using a volume-stable collagen matrix seeded with autologous gingival fibroblasts reported greater clinical attachment gain and greater reductions in recession depth and width than the identical unseeded matrix [130] (Table 8).

These studies provide proof of concept that introducing living cells can modify clinical scaffold performance, but they do not yet establish equivalence with autogenous grafting across all clinically relevant endpoints. None of these trials examined the survival, persistence, or secretory activity of the transplanted cells after implantation; outcomes were assessed by clinical or photogrammetric measurements of recession, root coverage, attachment level, keratinized tissue width, and gingival thickness. Improved clinical outcomes are therefore compatible with, but do not demonstrate, adaptive signaling by the seeded cells. Alternative explanations include extracellular matrix deposited by the cells during culture, a transient paracrine effect from cells that did not persist or altered handling and early wound stability of the pre-conditioned matrix. The only study in this group that labeled the transplanted cells is the preclinical dog study, in which stromal cells labeled with green fluorescent protein (GFP) were identified qualitatively at six weeks without quantification of their persistence [171] (Table 8). Adaptive cellular signaling should accordingly be regarded as a possible explanation rather than an established mechanism until cell fate is tracked in vivo. In particular, dimensional outcomes such as keratinized tissue width remain important unresolved endpoints.

The scaffold also remains a critical determinant of performance. Comparative preclinical studies demonstrate substantial differences among collagen matrices in porosity, stiffness, degradation, angiogenic behavior, and their interaction with gingival mesenchymal stromal cells [147]. A four-arm preclinical study similarly suggested different biological responses when a xenogeneic matrix was combined with autologous fibroblasts or gingiva-derived mesenchymal stromal cells [171]. A cell-seeded construct should be regarded as a material–cell pairing. Scaffold architecture determines cell retention, spatial distribution, nutrient diffusion, vascular access, mechanical protection, and ultimately whether implanted cells remain viable long enough to influence tissue formation.

The translational limitations are substantial. Autologous cell therapy may require biopsy, cell isolation and expansion, validated culture conditions, sterility testing, seeding, transport, and implantation within a controlled manufacturing pathway [172]. The biological responsiveness of the cells is therefore obtained at the cost of considerable manufacturing and regulatory complexity. Nevertheless, cell seeding differs fundamentally from the engineered responsive systems described above: its clinical feasibility has already been demonstrated at proof-of-concept level, whereas stimulus-responsive polymer interfaces for oral soft tissue augmentation remain predominantly preclinical.

### 8.6. Where the Field Currently Stands

**Figure 4:**
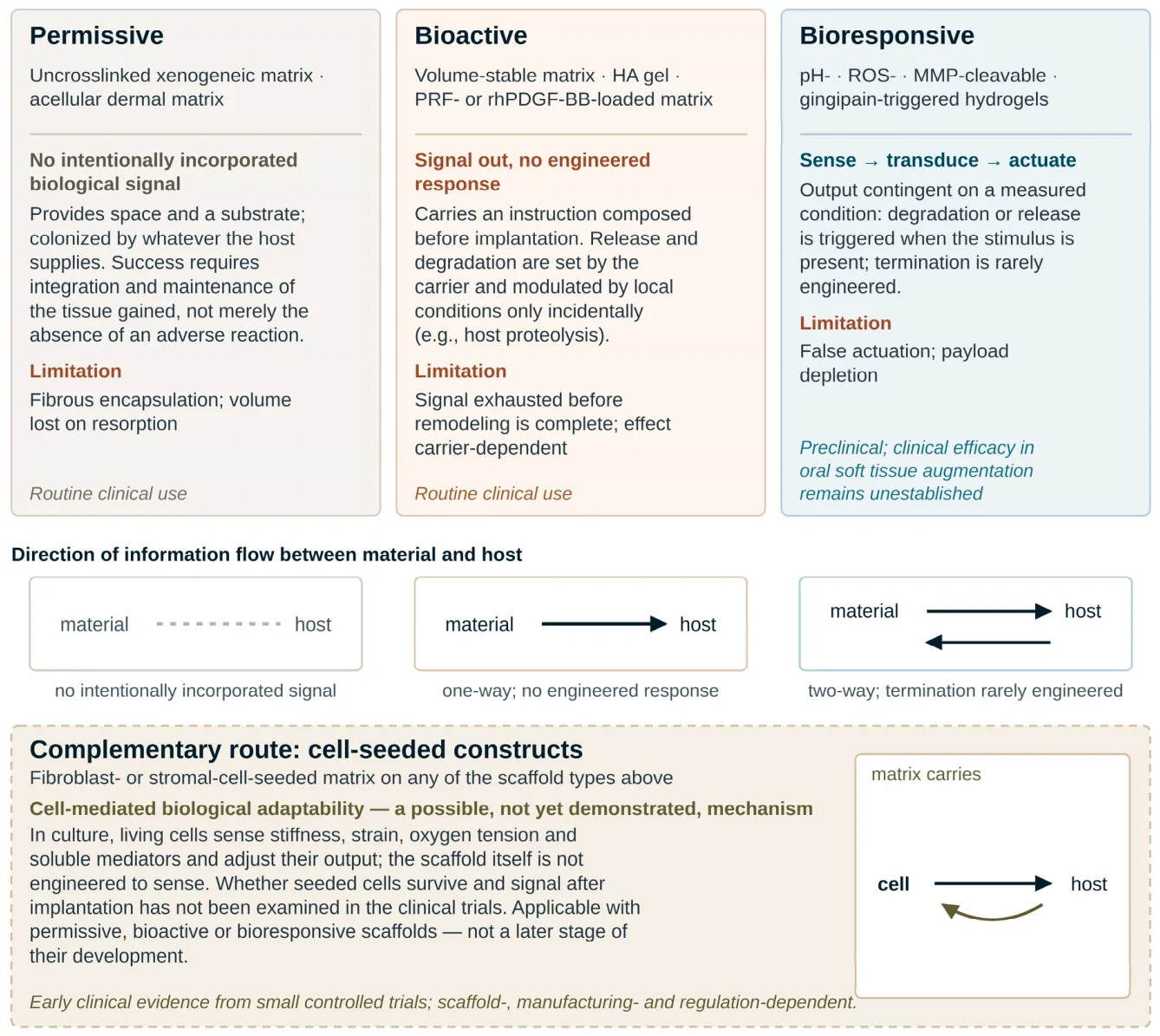
Three categories of material behavior in oral soft tissue augmentation—permissive, bioactive, and bioresponsive (Table 1)—and cell-seeded constructs as a complementary route in which adaptability may be supplied by living cells rather than by polymer chemistry; this cell-mediated mechanism is inferred from in vitro behavior and has not been demonstrated after implantation. The lower row summarizes the direction of information exchange between material and host. The categories describe design principles, not sequential stages of development. HA, hyaluronic acid; MMP, matrix metalloproteinase; PRF, platelet-rich fibrin; rhPDGF-BB, recombinant human platelet-derived growth factor-BB; ROS, reactive oxygen species.

Placing these strategies side by side (Figure 4), three levels of clinical maturity can be distinguished rather than two. First, optimized passive and bioactive systems are clinically available. Crosslinking, spatial architecture, surface functionalization, growth factors, and autologous concentrates can modify scaffold persistence, cellular interaction, and biological signaling. Their degradation and release profiles are influenced by local conditions—most obviously by host proteolysis—but only incidentally; none of these strategies couples material behavior to a defined biological signal by design.

Second, cell-seeded constructs may provide biological responsiveness and have reached early clinical evaluation. They introduce living cells that adapt to their environment in culture, but whether they survive and do so after implantation has not been examined; their performance remains dependent on the scaffold, and their translation is limited by manufacturing, logistics, cost, and regulatory requirements.

Third, engineered bioresponsive polymers provide stimulus-triggered degradation or release in principle but remain predominantly preclinical in this indication. Within the literature identified by the present search, no randomized clinical trial has established that a stimulus-responsive polymer interface improves oral soft tissue augmentation. Critical issues—including sterilization, trigger specificity, false activation, mechanical stability, payload depletion, and reproducibility—remain incompletely characterized [155].

The current evidence therefore does not justify presenting bioresponsive polymers as a clinically superior alternative to existing matrices. Their importance lies instead in providing a materials-science framework for addressing limitations that cannot be solved simply by increasing persistence or biological payload (Table 9). Cell-based and engineered responsiveness may ultimately prove complementary rather than competing strategies. Living cells could provide adaptive biological output, while the scaffold can provide controlled architecture, mechanical support, spatial organization, and potentially stimulus-dependent material behavior.

## 9. Translational Challenges and Research Priorities

The strategies described in Section 8 occupy different stages of translational development. Conventional biofunctionalized matrices are already used clinically, cell-seeded constructs have reached early clinical evaluation, whereas engineered stimulus-responsive polymer systems for oral soft tissue augmentation remain predominantly preclinical [155]. To our knowledge, and within the literature identified by the present search, no randomized clinical trial has demonstrated that a stimulus-responsive polymer interface improves clinical outcomes in oral soft tissue augmentation.

Translation of such systems will require more than demonstration of biological activity under controlled laboratory conditions. Responsive materials introduce additional requirements because their performance depends not only on composition and dose but also on the conditions under which activation occurs, the magnitude and duration of the response, and the behavior of the system after repeated or prolonged stimulation.

### 9.1. Translational Challenges

Regulatory classification

Responsive matrices may combine device, drug-delivery, and biological functions, and their regulatory pathway will depend on composition, mechanism of action, and intended clinical use. Unlike a conventional passive scaffold, a responsive system may require characterization of both its therapeutic output and the conditions under which that output is activated. Where viable cells are incorporated, additional requirements related to cell-based or advanced-therapy products may apply, including manufacturing control, traceability, sterility, viability, and comparability [173].

Manufacturing and scalability

Layer-by-layer assemblies, gradient structures, peptide-cleavable crosslinks, and multi-trigger systems may involve several sequential manufacturing steps. A laboratory-scale construct must therefore be translated into a reproducible process capable of controlling polymer composition, crosslink density, ligand concentration, payload loading, and stimulus-response characteristics between batches [93,174].

Sterilization

Sterilization is particularly important because responsive functionality may depend on chemically labile linkers, peptides, proteins, or polymer architectures. Conventional sterilization methods can alter collagen structure, mechanical properties, surface chemistry, and cellular interactions [78,79,175]. For responsive systems, sterilization must therefore be validated not only for sterility and structural integrity but also for preservation of the sensing and response mechanism.

Storage and shelf life

Responsive linkages, incorporated proteins, and particularly living cells may impose storage requirements substantially different from those of conventional collagen matrices. Shelf-life studies must establish whether mechanical properties, biological activity, trigger sensitivity, and response magnitude remain stable throughout storage [176]. Cell-containing products introduce additional constraints related to viability, transport, and potentially cold-chain logistics.

Mechanical performance

Chemical sophistication does not remove the basic surgical requirements of a soft tissue scaffold. A clinically useful material must tolerate hydration, trimming, suturing, flap pressure, wound contraction, and functional loading. Responsive behavior must be introduced without compromising the mechanical properties required for placement and early stabilization.

Trigger specificity and threshold calibration

A major challenge is distinguishing pathological signals from physiological fluctuations. Oral tissues are exposed to changes in temperature, pH, salivary composition, microbial activity, and mechanical loading. A system responsive to pH, ROS, enzymes, or mechanical stress must demonstrate a defined activation threshold, an appropriate off-state, and acceptable resistance to false activation under clinically relevant conditions.

Dose quantification and response reproducibility

Conventional delivery systems already face challenges in defining the effective local dose. Responsive systems add another variable because the delivered amount may depend intentionally on stimulus intensity and duration. Characterization should therefore include not only total payload and cumulative release but also stimulus–response curves, on/off ratios, repeated activation behavior, and inter-batch reproducibility.

Payload depletion and duration of function

Responsiveness determines when a finite payload is released, but it does not eliminate eventual depletion. This distinction is particularly important at the peri-implant interface, where biological maintenance may be required for years whereas most drug-delivery systems operate over days to months. Responsive materials may be most realistic as interventions during defined healing or inflammatory windows unless their function can be renewed, recharged, or based on recruitment of endogenous biological signals.

Biosafety

More complex systems may introduce degradation products, nanoparticles, inorganic components, reactive intermediates, or permanently retained constituents that are not present in conventional collagen matrices. Their local and systemic safety must be evaluated according to the specific chemistry used rather than assumed from the general concept of responsiveness.

Preclinical models

Many proof-of-concept studies use simplified microbial cultures, isolated cell systems, chorioallantoic membrane assays, or small-animal models [177]. These approaches are valuable for mechanistic testing but do not reproduce the complete geometry, microbial complexity, mechanical environment, or long-term biological demands of a transmucosal dental interface [3,5]. Preclinical models should increasingly reproduce mixed-species biofilms, inflammatory conditions, relevant tissue thicknesses, and clinically realistic mechanical loading.

Clinical endpoints

Mucosal thickness and keratinized tissue width remain important dimensional outcomes, but volumetric assessment methods differ substantially among studies. Aesthetic outcomes and patient-reported measures should also be retained because reduced morbidity is one of the principal advantages of substitute materials.

Cost-effectiveness

The relevant clinical comparator remains autogenous connective tissue grafting, which is effective, widely available, and does not require complex manufacturing [72]. A responsive or cell-based system will need to demonstrate a clinically meaningful advantage—whether in morbidity, dimensional stability, predictability, maintenance, or workflow—to justify additional complexity and cost.

### 9.2. Research Priorities

Based on these translational challenges, several research priorities emerge.

1. Define and report stimulus-response characteristics under clinically relevant conditions. Studies should report activation thresholds, response magnitude, off-state leakage, reversibility where applicable, and false-activation behavior under repeated pH, temperature, enzymatic, microbial, and mechanical challenges.
2. Establish a minimum reporting dataset for responsive materials. At minimum, studies should report the trigger, activation threshold, response mechanism, payload per unit mass or area, cumulative release, on/off ratio, repeated-response behavior, degradation profile, and post-depletion state. Without such standardization, comparison between systems will remain difficult.
3. Use biologically relevant models. Candidate materials should be tested in mixed-species biofilm systems, inflamed-tissue models, and models reproducing clinically relevant tissue geometry rather than relying exclusively on single-species or isolated-cell assays.
4. Match the design objective to the intended biological window. A material intended to improve early vascularization has different requirements from one intended to control inflammatory episodes or support long-term transmucosal maintenance. Not every responsive system needs indefinite activity, but the intended duration of function should be defined explicitly.
5. Prioritize designs with realistic translational pathways. Injectable systems, membranes, or matrices that modify an established clinical workflow may offer a more feasible route to translation than highly complex permanently implanted responsive interfaces.
6. Report long-term dimensional and patient-centered outcomes. Studies of augmentation materials should include follow-up beyond the early healing period, ideally at 12 months or longer, and should incorporate volumetric, aesthetic, and patient-reported outcomes alongside conventional periodontal measures.

### 9.3. Future Prospects: Personalized Tissue-Engineered Constructs

Among the strategies considered in this review, cell-seeded scaffolds represent a distinct translational pathway because they could introduce biological responsiveness without requiring the polymer itself to perform engineered molecular sensing. Unlike stimulus-responsive polymer systems, cell-containing constructs have already reached early clinical evaluation in oral soft tissue applications.

The concept is not simply to add cells to an existing matrix, but to create a material–cell construct in which scaffold properties are selected specifically to support the biological requirements of the seeded population [178]. Patient-derived gingival fibroblasts can respond to stiffness, oxygen tension, mechanical strain, extracellular matrix composition, and soluble mediators and can modify their proliferation, migration, matrix synthesis, and secretory profile accordingly. Autologous oral mucosal equivalents generated from biopsy-derived cells have also been applied clinically [179,180].

A future tissue-engineered construct could therefore integrate three components.

First, a cellular component would provide living, environmentally responsive biological activity. Depending on the intended function, this could include gingival fibroblasts alone or combinations with other relevant cell populations. Second, a characterized scaffold would provide the architecture, mechanical support, and degradation profile required for cell retention, vascularization, and tissue formation. Consequently, scaffold selection should be based on measured properties. Third, optional biofunctionalization could provide additional biological signaling where justified. As demonstrated by the carrier-dependent effects discussed in Section 6, the biological agent should be selected together with the scaffold rather than considered an independent therapeutic component. This represents a shift from viewing the scaffold as an off-the-shelf substitute toward viewing it as one component of a regenerative system. A matrix that performs adequately as an acellular filler may not necessarily be the optimal carrier for living fibroblasts, and the properties required for root coverage may differ from those required for volume augmentation or peri-implant tissue formation.

The principal unresolved questions are increasingly materials-based. Which scaffold architecture supports uniform cellular distribution rather than superficial colonization? Which pore dimensions and interconnectivity permit vascular ingrowth rapidly enough to sustain seeded cells? Which degradation profile allows tissue replacement without premature loss of volume? Which mechanical properties protect cells during hydration, trimming, suturing, and early wound contraction? These questions should be answered through direct material characterization and material–cell testing before clinical implantation rather than inferred retrospectively from surgical outcomes.

Personalized cell-seeded constructs are not yet a routine alternative to autogenous grafting, and their manufacturing, regulatory, logistical, and economic burdens remain substantial. Nevertheless, they provide a clinically testable model of biological adaptability—provided that the fate of the transplanted cells is tracked to confirm that this mechanism operates in vivo—and may also serve as a bridge between conventional passive scaffolds and more complex engineered bioresponsive systems.

## 10. Conclusions

Polymer-based substitutes have substantially expanded the possibilities for oral soft tissue augmentation by reducing donor-site morbidity, simplifying surgical procedures, and improving patient acceptance. However, current substitutes do not consistently reproduce the gains in mucosal thickness and keratinized tissue width achieved with autogenous connective tissue across all materials, indications, and follow-up periods. In several direct comparisons involving collagen-based matrices, the difference in soft tissue thickness gain favors autogenous grafts by approximately 0.3–0.4 mm, although this magnitude is not universal and varies according to the scaffold and clinical setting.

The available evidence indicates that this difference cannot be attributed to a single material property or biological mechanism. Scaffold architecture, mechanical behavior, vascular and cellular colonization, degradation kinetics, wound contraction, biological signaling, and the interaction between the carrier and the host may all contribute to the final outcome.

Crosslinking, spatial organization, growth-factor delivery, platelet concentrates, and other biofunctionalization strategies can improve selected aspects of scaffold performance, but none of them deliberately couples material behavior to the changing requirements of healing. Their behavior is influenced by local tissue conditions, but through incidental processes such as host proteolysis rather than through an engineered response mechanism (Section 7.9).

The clinical evidence reviewed here also suggests that the carrier is an integral component of any biological intervention. The same biological agent may produce different outcomes when delivered from different scaffolds, emphasizing that bioactive molecules should be evaluated as part of a material–biology system rather than as independent therapeutic entities.

Cell-seeded constructs provide a distinct approach by introducing living cells that may respond dynamically to their local environment. Early clinical studies support their clinical feasibility but have not examined the survival or function of the transplanted cells, so adaptive cellular signaling remains a possible rather than an established mechanism; current evidence also remains insufficient to establish equivalence or superiority to autogenous grafts for thickness, keratinized tissue width, and volume stability. Autogenous grafts remain the reference standard, with the connective tissue graft primarily used for root coverage and tissue thickening and the free gingival graft for increasing keratinized tissue width.

Engineered bioresponsive polymers offer another potential strategy by allowing selected material functions to change in response to biological, enzymatic, microbial, chemical, or mechanical stimuli. Their relevance lies in the possibility of coupling material behavior more closely to the evolving state of the wound. However, their application to oral soft tissue augmentation remains predominantly preclinical, and no clinical evidence currently demonstrates superiority over conventional substitutes or autogenous grafts.

Future development should therefore focus not simply on increasing scaffold persistence or adding more potent biological signals, but on better coordinating material architecture, degradation, cellular colonization, biological signaling, and the temporal requirements of healing. Particular attention should be given to well-characterized material–cell and material–bioactive agent combinations, stimulus–response specificity, reproducible manufacturing, long-term dimensional outcomes, and clinically meaningful patient-centered endpoints.

The most promising direction may therefore be a transition from viewing soft tissue substitutes as isolated passive scaffolds toward viewing them as integrated material–biology systems. Whether this integration is achieved through improved passive design, living cellular components, engineered bioresponsiveness, or combinations of these strategies will require direct clinical testing. At present, bioresponsiveness should be regarded as a compelling design concept and research direction rather than an established clinical solution.

## Figures and Tables

**Table 1:** Comparison of permissive, bioactive, and bioresponsive material concepts relevant to oral soft tissue augmentation. Biocompatibility is a prerequisite for all three categories and is therefore listed as a shared requirement rather than as a category.

Criterion	Passive/Permissive	Bioactive/Instructive	Bioresponsive/Biointeractive	Ref.
Prerequisite	Biocompatibility—ISO 10993-1:2025: biological safety established through a risk management process rather than a fixed test set; required for all three categories			[23]
Defining question	Does it provide space and a substrate without directing the response?	Does it elicit a defined response?	Does it respond to what is happening?	—
Definition	Biocompatible scaffold that permits host colonization without a programmed biological instruction	Designed to elicit or modulate biological activity within the bioactivity zone	Sensitive to a biological signal or pathological state and actuated by it	[13]
Verification	Space maintenance; host cell infiltration; absence of encapsulation	Domain-specific endpoints: cell adhesion, matrix deposition, antimicrobial activity	Trigger identity and threshold; on/off ratio; false-activation rate	—
Information flow	No intentionally programmed biological instruction	Predominantly one-way; pre-programmed instruction	State-dependent; an environmental stimulus modifies material behavior or output	[17,18]
Failure mode	Fibrous encapsulation	Signal delivered at the wrong time or after the need has passed	Failure to actuate, or actuation in response to noise	[16,20]
Soft tissue examples	Uncrosslinked xenogeneic matrix; acellular dermal matrix	Hyaluronic acid; concentrate- or growth-factor-biofunctionalized matrix	MMP-cleavable and gingipain-responsive hydrogels (preclinical)	[24,25,26,27]
Clinical status	In routine use	In routine use	No clinical trial identified in the present search	—

**Table 2:** Polymer-based and autogenous platforms for oral soft tissue augmentation. Platforms are ordered from autogenous tissue, which defines the performance target, through the xenogeneic and allogeneic matrices developed to replace it, to synthetic and injectable systems. Degradation timeframes are indicative and vary with product, tissue source, crosslinking, and site.

Platform	Composition and Origin	Principal Indication	Degradation	Principal Advantage	Principal Limitation	Ref.
Connective tissue graft	Autogenous subepithelial connective tissue	Thickness and volume gain; recession coverage	Integrates and remodels with host; no resorption endpoint	Living tissue; integrates, revascularizes, and remodels with the host	Donor site morbidity; finite supply; operating time	[8,10,72]
Free gingival graft	Autogenous keratinized masticatory mucosa	Keratinized mucosa width	As above	Most effective for keratinized width	Donor morbidity; color and texture mismatch	[10,84]
Xenogeneic collagen matrix	Porcine collagen-rich tissues; processing and crosslinking are product-dependent	Thickness gain; keratinized width	Enzymatic; persistence is product- and processing-dependent	No donor site; unlimited supply; good aesthetics	Volumetric loss during remodeling	[74,75,85]
Volume-stable collagen matrix	Porcine collagen, crosslinked	Thickness gain at implant sites	Enzymatic, prolonged by crosslinking	Improved dimensional retention	Potential trade-off between prolonged persistence and tissue integration, depending on crosslinking chemistry and density	[76,86]
Acellular dermal matrix	Allogeneic dermis, decellularized	Recession coverage; thickness gain	Enzymatic; remodeled by host cells	Retains basement membrane; early performance close to graft	Allogeneic sourcing; batch variability; cost	[77,84]
Synthetic biodegradable polymers	PLGA, PLA, PCL, PLCL	Barrier membranes; composite carriers	Bulk hydrolysis; weeks to months, tunable	High pre-implantation tunability of degradation and architecture	Acidic degradation products; hydrolytic profile set by composition and molecular weight, largely independent of local enzymatic activity	[80,81]
Hydrogels	Hyaluronic acid; chitosan; PEG networks	Papillae; irregular defects; injectable adjunct	Enzymatic or dissolution-dependent; days to weeks	Conforms to sites no matrix can reach	Mechanically weak; rapid in vivo degradation	[25,48]

**Table 3:** Biofunctionalization strategies for oral soft tissue matrices. Strategies are distinguished by the nature of the payload and by the manner of its association with the scaffold, rather than by a single ordered scale. Temporal control and environmental responsiveness are reported separately, since a strategy may permit considerable control over the release profile while remaining insensitive to the state of the site.

Strategy	Payload	Association with Scaffold	Signal Presentation/Release	Temporal Control	Environmental Responsiveness	Principal Limitation	Clinical Status	Ref.
Adsorption/soak-loading	Defined exogenous protein	Non-oriented physical adsorption; pore entrapment	Predominantly early release governed by desorption and diffusion	Limited; profile set by carrier design, modulated incidentally by local degradation	None	Loaded dose not readily quantified or reproduced	In routine clinical use	[44,87]
Autologous concentrate loading	Multi-factor, patient-derived preparation	Physical loading into the scaffold	Release over approximately 10–14 days	Limited; determined by preparation and scaffold	None	Composition and delivered dose not standardized	In routine clinical use	[88,89,90]
Cell seeding	Living autologous cells	Cellular colonization of the scaffold before implantation	Cell-derived signaling, in principle continuous while cells survive	Determined by the cells rather than by the material; not demonstrated after implantation	Cell-mediated, possible but not demonstrated in vivo; not engineered into the material	Manufacture, viability logistics and advanced-therapy classification	Early clinical evidence	[91,92]
Affinity tethering	Heparin-binding growth factors	Reversible electrostatic association with heparin or GAG mimetics	Sustained release governed by a binding equilibrium	Moderate; release follows the local binding equilibrium, without an engineered trigger	None unless a responsive component is incorporated	Finite payload; release trigger remains non-specific	Preclinical	[94,95,97]
Covalent immobilization	Peptides and stable protein motifs	Irreversible covalent bond via a short linker	Persistent surface presentation	High spatial control; limited temporal adaptability	None unless a responsive linker is incorporated	Possible conformational change or active-site masking	Preclinical	[98,99,100]

**Table 4:** Comparative clinical evidence for soft tissue substitutes and autogenous grafts, by endpoint and site. Quantitative differences are presented individually rather than pooled, because their magnitude varies according to material, indication, endpoint, and study design. Root coverage is not included because comparative root-coverage evidence at natural teeth is presented in Section 5.5 and Section 6. The final column records the unit of analysis and the principal limitations of each source as reported, or as far as they could be established from the report available to us (Section 6.7).

Study Design	Comparison	Site	Endpoint	Result	Difference Favoring Graft	Ref.	Unit of Analysis; Main Limitations
Systematic review and meta-analysis, 19 RCTs and CCTs	Collagen matrix vs. connective tissue graft	Teeth and implants (pooled)	Mucosal thickness gain	Graft superior	0.32 mm (95% CI 0.16–0.49)	[102]	Study-level pooling; the 19 reports include four from one 20-patient trial; teeth and implants pooled; three trials compared a matrix with a free gingival graft
Same analysis	Collagen matrix vs. connective tissue graft	Teeth and implants (pooled)	Keratinized tissue width gain	Graft superior	0.46 mm (95% CI 0.02–0.89)	[102]	As above; the pooled keratinized tissue estimate is reported inconsistently within this review (0.46 mm in the abstract, 0.62 mm in the results)
RCT, *n* = 60, augmentation at implant uncovering	Xenogeneic collagen matrix vs. graft	Implant	Buccal thickness at 6 months	CTG: 1.2 ± 0.3 mm; matrix: 0.9 ± 0.2 mm (*p* = 0.0001)	0.30 mm (95% CI 0.2–0.5)	[104]	One implant per patient; patient = unit; 2 of 60 lost, intention-to-treat; single center; needle-and-caliper thickness (examiner ICC 0.87); 6 months
RCT, *n* = 30, augmentation at implant placement	Xenogeneic collagen matrix vs. graft	Implant	Thickness gain at 3 months	CTG: 1.55 ± 0.11 mm; matrix: 1.18 ± 0.11 mm (*p* = 0.016)	0.37 mm (95% CI 0.07–0.66)	[105]	Parallel groups, 15 per arm, all completed; 3-month follow-up; thickness by superimposition of scanned models on cone-beam CT, with repeated measurement points per patient modeled by random intercepts
Multicenter non-inferiority RCT, *n* = 100 randomized (98 completed), 12 months	Volume-stable collagen matrix vs. graft	Implant	Peri-implant mucosal thickness	CTG: 1.0 ± 0.75 mm; matrix: 0.66 ± 0.58 mm (*p* = 0.002)	0.37 mm (95% CI 0.13–0.61); non-inferiority not demonstrated	[86]	Patient = unit (ANCOVA); 100 randomized, 98 completed (one lost per group); 4 centers; needle-and-caliper thickness, intraoral scans for volume; 12 months
Meta-analysis, immediate placement	Acellular dermal matrix vs. graft	Implant (immediate placement)	Thickness gain at 12 months	CTG: 0.46 ± 0.53 mm; ADM: 0.33 ± 0.44 mm	0.13 mm, not significant; high heterogeneity	[107]	5 studies, 3 pooled for thickness; high heterogeneity; trial set independent of the collagen-matrix reviews
Network meta-analysis, 23 studies, 573 sites	All materials	Implant	Thickness at 180 and 360 days	Graft ranked best at both intervals	—	[84]	23 studies (16 in the network, including 5 controlled non-randomized trials); rankings rather than effect sizes; includes the same principal trials as [85,102,103,106]

**Table 6:** Clinical evidence for biofunctionalization of soft tissue matrices. Studies are grouped by biological agent and carrier; randomized comparisons against the identical unfunctionalized carrier are distinguished from comparisons involving different surgical procedures and from uncontrolled case series. The final column records the unit of analysis, whether multiple sites per patient were modeled or treated as independent, attrition, and the measurement method, as far as these could be established from the report available to us (Section 6.7).

Agent	Carrier	Design	Follow-Up	Principal Outcome	Interpretation Relative to the Carrier	Ref.	Unit of Analysis; Main Limitations
rhPDGF-BB	Cross-linked volume-stable collagen matrix	Triple-blind RCT, placebo-controlled, n = 30	6 months	Higher mean root coverage, greater gingival thickness, volume and aesthetic score	Significant added benefit at 6 months	[121]	Parallel arms; 91 sites in 30 patients analyzed with mixed-effects models accounting for multiple sites per patient; no dropouts; probe, intraoral-scan superimposition and ultrasonography; no connective tissue graft arm
rhPDGF-BB	Same matrix, same cohort	3-year follow-up, n = 26	3 years	Both groups stable over three years; changes from 6 months to 3 years did not differ between groups, while the early advantages in root coverage, recession reduction, attachment gain, volume and aesthetics were maintained over the observation period	Early benefit maintained at 3 years; stability in both groups	[123]	26 of 30 patients (80 sites) at 3 years, attrition 13%; mixed-effects models accounting for multiple sites per patient; probe, digital-model superimposition and ultrasonography; no connective tissue graft arm
rhPDGF-BB	Acellular dermal matrix	Split-mouth comparison with randomized side allocation, 40 defects, 17 patients	6 months	Root coverage 69.0% with growth factor versus 76.7% with saline	No significant added benefit	[124]	Split-mouth; patient = unit with teeth nested within patients in linear mixed models; 6 months; probe on stent to 0.5 mm; attrition not reported
rhFGF-2	Absorbable collagen scaffold	Single-arm, open-label first-in-human study, 21 patients, 34 sites	6 months	Reduced recession depth and greater keratinized width at 6 months compared with baseline	No comparator; added effect of the growth factor cannot be isolated	[128]	Single arm; 34 sites from 21 patients, several contributing 2–3 sites, analyzed as independent; 3 sites lost; probe only; no thickness measurement or histology
FGF-2	Bioresorbable collagen membrane	Parallel-group RCT, n = 24, one recession per patient	6 months	Greater recession reduction, keratinized width, attachment gain and phenotype change	Significant added benefit	[129]	Parallel groups, one recession per patient (12 per group); 6 months; probe; between-group test not specified
Autologous gingival fibroblasts	Volume-stable collagen matrix	Split-mouth RCT, 20 sites, 10 patients	6 months	Greater attachment gain and reduction in recession depth and width	Significant benefit versus identical unseeded matrix; proof-of-concept clinical evidence	[130]	Split-mouth, 20 sites in 10 patients (one site per side), paired Wilcoxon analysis; no dropouts; periodontal probe; 6 months; keratinized tissue width not significantly different; fate of seeded cells not examined
Polynucleotides + hyaluronic acid	Volume-stable collagen matrix	Prospective case series, n = 16	12 months	High mean and complete root coverage; gingival thickness gain	Added effect cannot be isolated; no matrix-only comparator included	[131]	Case series without comparator; multiple recessions per patient
Platelet-rich plasma	Acellular dermal matrix	Randomized split-mouth, 84 recessions, 12 patients	12 months	Higher mean root coverage with plasma	Significant added benefit in a single trial	[127]	Split-mouth; 84 teeth in 12 patients analyzed as independent observations without clustering adjustment; 2 of 14 patients lost; probe on stent, caliper for keratinized width
Injectable PRF	Volume-stable collagen matrix	Three-arm RCT, n = 75	6 months	No difference in recession reduction; marginal thickness signal	No significant added benefit	[125]	Parallel groups, 25 per arm, one recession per patient, all 75 analyzed; 6 months; probe on stent, thickness by endodontic spreader and caliper; single masked calibrated examiner
Liquid PRF	Volume-stable collagen matrix	Split-mouth RCT, 25 enrolled, 24 analyzed	6 months	No difference in any clinical, aesthetic or patient-reported outcome	No significant added benefit	[126]	Split-mouth; one tooth per side (deepest recession) entered the paired Wilcoxon analysis, 48 paired assessments in 24 patients; 1 of 25 excluded for incomplete follow-up; probe and needle-with-stop thickness; 6 months
Enamel matrix derivative	Xenogeneic collagen matrix	Four-arm RCT, n = 68	6 months	Combination no better than either component alone	No clear additional benefit of the combination	[136]	Parallel groups, one recession per patient (17 per arm); no participants lost to follow-up; 6 months; probe, two calibrated masked examiners
Enamel matrix derivative	Acellular dermal matrix	Meta-analysis, 4 RCTs, 58 patients, 194 defects	6–12 months	Root coverage difference not significant	No significant added benefit	[137]	4 split-mouth RCTs; 194 defects from 58 patients pooled without a described paired analysis; 6–12 months

**Table 8:** Cell-seeded scaffolds for gingival recession and soft tissue augmentation. Inclusion is restricted to controlled studies; descriptive case series are excluded. Clinical and preclinical investigations are identified separately, since they support different claims; the comparator column records what each seeded construct was tested against—the identical unseeded scaffold, a flap alone, or an autogenous graft; and the final column records whether the survival or function of the transplanted cells was examined after implantation (Section 8.5).

Study	Carrier	Cells	Design	Comparator	Principal Outcome	Evidence Level/Setting	Ref.	Survival or Function of Seeded Cells After Implantation
Köseoğlu 2013	Collagen membrane	Autologous gingival fibroblasts	Split-mouth randomized controlled pilot, 11 patients, 22 sites (one pair per patient), 12 months	Identical unseeded collagen membrane under a coronally advanced flap	Significantly greater percentage root coverage in the seeded group; no difference in gingival thickness	Clinical—split-mouth randomized controlled pilot	[169]	Not examined; clinical (probe, endodontic spreader for thickness) and photogrammetric outcomes only
Milinkovic 2015	Collagen matrix	Autologous fibroblast cell culture	Split-mouth randomized comparison, 18 patients, 48 teeth, 12 months	Connective tissue graft	Both effective; graft superior for keratinized tissue width only	Clinical—split-mouth randomized comparison	[170]	Not examined in vivo; viability and fibroblast identity confirmed in culture only
Sanchez 2022	Xenogeneic collagen matrix	Autologous fibroblasts; gingiva-derived mesenchymal stromal cells	Four-arm preclinical split-mouth pilot, dehiscence defects in 7 dogs (56 sites; tooth = unit of analysis), 2 and 6 weeks	Flap alone; matrix alone	Both cell types reduced inflammation and produced variable new cementum and bone; greatest recession reduction with mesenchymal cells, not statistically significant	Preclinical—dog dehiscence model	[171]	Stromal cells GFP-labeled; GFP-positive cells identified qualitatively by immunohistochemistry at 6 weeks in selected sections, persistence not quantified; fibroblasts not labeled
Koteneva 2025	Three xenogeneic scaffolds	Human gingival mesenchymal stromal cells	Preclinical: mechanical, micro-CT, cytocompatibility, CAM, rabbit	Scaffold alone	Porosity 78.5%, 64.3% and 33.2%; degradation from complete resorption to near-complete retention; lamina propria thicker than intact gingiva with all scaffolds at 14 days, with no significant difference between seeded and unseeded scaffolds (trend for one pairing, *p* = 0.064); cells reduced vessel density with the most porous scaffold	Preclinical—in vitro, CAM and rabbit	[147]	Not examined; no labeling or tracking of the human cells; seeded and unseeded scaffolds compared at a single 14-day endpoint
Sankar 2026	Volume-stable collagen matrix	Autologous gingival fibroblasts	Split-mouth randomized controlled trial, 10 patients, 20 sites	Identical unseeded matrix	Significantly greater attachment gain and reduction in recession depth and width at all intervals	Clinical—split-mouth randomized trial	[130]	Not examined; clinical outcomes only

**Table 9:** Translation of the principal limitations of conventional soft tissue substitutes into functional requirements and potential material-design strategies. Proposed responsive approaches represent research directions and should not be interpreted as clinically validated solutions.

Limitation	Established in	Functional Requirement	Potential Design Response	Current Evidence
Volumetric instability during remodeling	Section 5; Section 7.1	Maintain space until replacement tissue matures	Tuned degradation and mechanics; potentially responsive degradation	Clinical limitation established; responsive solution preclinical
Degradation not deliberately coupled to tissue maturation	Section 7.2	Better coordination of material loss with tissue replacement	Enzyme- and MMP-responsive crosslinks	Preclinical
Limited state sensing and feedback	Section 7.3	State-dependent actuation with defined thresholds	pH-, ROS-, enzyme- and mechano-responsive systems	Preclinical
Dependence on host cellular colonization	Section 7.4	Early and sufficient cellular and vascular colonization	Cell seeding; prevascularization; hypoxia-responsive delivery	Early clinical evidence for cell seeding; responsive delivery preclinical
Single predefined architecture	Section 7.5	Spatially differentiated functions	Layered, gradient and 4D constructs	Predominantly preclinical
Long-term maintenance challenge	Section 7.6	Durable or repeatable function and formation of stable host tissue	Feedback-controlled, replenishable or endogenous-signal-recruiting systems	Conceptual and preclinical
Sourcing and batch variability	Section 7.7	Reproducible composition and performance	Defined synthetic and composite systems; manufacturing quality control	Translational requirement

## Data Availability

No new data were created or analyzed in this study. Data sharing is not applicable to this article.

## Abbreviations

The following abbreviations are used in this manuscript:

A-PRF	advanced platelet-rich fibrin
Alb-PRF	albumin-enriched platelet-rich fibrin
CAM	chorioallantoic membrane
C-PRF	concentrated platelet-rich fibrin
CI	confidence interval
EGF	epidermal growth factor
EMD	enamel matrix derivative
FGF-2	fibroblast growth factor-2
GAG	glycosaminoglycan
GFP	green fluorescent protein
IGF-1	insulin-like growth factor-1
i-PRF	injectable platelet-rich fibrin
L-PRF	leukocyte- and platelet-rich fibrin
MMP	matrix metalloproteinase
PCL	polycaprolactone
PDGF	platelet-derived growth factor
PDGF-AB	platelet-derived growth factor, AB isoform
PDGF-BB	platelet-derived growth factor, BB isoform
PEG	poly(ethylene glycol)
PERK	protein kinase R-like endoplasmic reticulum kinase
PLA	polylactic acid
PLCL	poly(L-lactide-co-caprolactone)
PLGA	poly(lactic-co-glycolic acid)
PRF	platelet-rich fibrin
RCT	randomized controlled trial
RGD	arginine–glycine–aspartic acid peptide motif
rhPDGF-BB	recombinant human platelet-derived growth factor-BB
ROS	reactive oxygen species
SDF-1	stromal cell-derived factor-1
TGF-β	transforming growth factor-beta
VEGF	vascular endothelial growth factor

## References

[1] Cortellini P., Bissada N.F. (2018). Mucogingival conditions in the natural dentition: Narrative review, case definitions, and diagnostic considerations. J. Periodontol..

[2] Kim D.M., Neiva R. (2015). Periodontal soft tissue non-root coverage procedures: A systematic review from the AAP Regeneration Workshop. J. Periodontol..

[3] Berglundh T., Lindhe J., Ericsson I., Marinello C.P., Liljenberg B., Thomsen P. (1991). The soft tissue barrier at implants and teeth. Clin. Oral Implants Res..

[4] Guo T., Gulati K., Arora H., Han P., Fournier B., Ivanovski S. (2021). Race to invade: Understanding soft tissue integration at the transmucosal region of titanium dental implants. Dent. Mater..

[5] Sculean A., Gruber R., Bosshardt D.D. (2014). Soft tissue wound healing around teeth and dental implants. J. Clin. Periodontol..

[6] Suárez-López Del Amo F., Lin G.H., Monje A., Galindo-Moreno P., Wang H.L. (2016). Influence of Soft Tissue Thickness on Peri-Implant Marginal Bone Loss: A Systematic Review and Meta-Analysis. J. Periodontol..

[7] Thoma D.S., Naenni N., Figuero E., Hämmerle C.H.F., Schwarz F., Jung R.E. (2018). Effects of soft tissue augmentation procedures on peri-implant health or disease: A systematic review and meta-analysis. Clin. Oral Implants Res..

[8] Zucchelli G., Tavelli L., McGuire M.K., Rasperini G., Feinberg S.E., Wang H.L., Giannobile W.V. (2020). Autogenous soft tissue grafting for periodontal and peri-implant plastic surgical reconstruction. J. Periodontol..

[9] Thoma D.S., Benić G.I., Zwahlen M., Hämmerle C.H., Jung R.E. (2009). A systematic review assessing soft tissue augmentation techniques. Clin. Oral Implants Res..

[10] Griffin T.J., Cheung W.S., Zavras A.I., Damoulis P.D. (2006). Postoperative complications following gingival augmentation procedures. J. Periodontol..

[11] Williams D.F. (2008). On the mechanisms of biocompatibility. Biomaterials.

[12] Anderson J.M., Rodriguez A., Chang D.T. (2008). Foreign body reaction to biomaterials. Semin. Immunol..

[13] Ghasemi-Mobarakeh L., Kolahreez D., Ramakrishna S., Williams D. (2019). Key Terminology in Biomaterials and Biocompatibility. Curr. Opin. Biomed. Eng..

[14] Hench L.L., Splinter R., Allen W.C., Greenlee T.K. (1971). Bonding mechanisms at the interface of ceramic prosthetic materials. J. Biomed. Mater. Res..

[15] Hench L.L., Polak J.M. (2002). Third-generation biomedical materials. Science.

[16] Williams D.F. (2021). Biocompatibility pathways and mechanisms for bioactive materials: The bioactivity zone. Bioact. Mater..

[17] Lu Y., Aimetti A.A., Langer R., Gu Z. (2017). Bioresponsive materials. Nat. Rev. Mater..

[18] Stuart M.A.C., Huck W.T.S., Genzer J., Müller M., Ober C., Stamm M., Sukhorukov G.B., Szleifer I., Tsukruk V.V., Urban M. (2010). Emerging applications of stimuli-responsive polymer materials. Nat. Mater..

[19] Mura S., Nicolas J., Couvreur P. (2013). Stimuli-responsive nanocarriers for drug delivery. Nat. Mater..

[20] Yu J., Zhang Y., Yan J., Kahkoska A.R., Gu Z. (2018). Advances in bioresponsive closed-loop drug delivery systems. Int. J. Pharm..

[21] Guiseppi-Elie A. (2014). Targeting homeostasis in drug delivery using bioresponsive hydrogel microforms. Int. J. Pharm..

[22] Montoya C., Du Y., Gianforcaro A.L., Orrego S., Yang M., Lelkes P.I. (2021). On the road to smart biomaterials for bone research: Definitions, concepts, advances, and outlook. Bone Res..

[23] (2025). Biological Evaluation of Medical Devices—Part 1: Requirements and General Principles for the Evaluation of Biological Safety within a Risk Management Process.

[24] Tavelli L., McGuire M.K., Zucchelli G., Rasperini G., Feinberg S.E., Wang H.L., Giannobile W.V. (2020). Extracellular matrix-based scaffolding technologies for periodontal and peri-implant soft tissue regeneration. J. Periodontol..

[25] Eliezer M., Imber J.C., Sculean A., Pandis N., Teich S. (2019). Hyaluronic acid as adjunctive to non-surgical and surgical periodontal therapy: A systematic review and meta-analysis. Clin. Oral Investig..

[26] Zhou Y., Liu J., Xue P., Zhang J. (2023). Collagenase-responsive hydrogel loaded with GSK2606414 nanoparticles for periodontitis treatment through inhibiting inflammation-induced expression of PERK of periodontal ligament stem cells. Pharmaceutics.

[27] Liu S., Wang Y.N., Ma B., Shao J., Liu H., Ge S. (2021). Gingipain-responsive thermosensitive hydrogel loaded with SDF-1 facilitates in situ periodontal tissue regeneration. ACS Appl. Mater. Interfaces.

[28] Barrientos S., Stojadinovic O., Golinko M.S., Brem H., Tomic-Canic M. (2008). Growth factors and cytokines in wound healing. Wound Repair Regen..

[29] Werner S., Grose R. (2003). Regulation of wound healing by growth factors and cytokines. Physiol. Rev..

[30] Johnson K.E., Wilgus T.A. (2014). Vascular Endothelial Growth Factor and Angiogenesis in the Regulation of Cutaneous Wound Repair. Adv. Wound Care.

[31] Bainbridge P. (2013). Wound healing and the role of fibroblasts. J. Wound Care.

[32] Werner S., Krieg T., Smola H. (2007). Keratinocyte-fibroblast interactions in wound healing. J. Investig. Dermatol..

[33] Mitchell A.C., Briquez P.S., Hubbell J.A., Cochran J.R. (2016). Engineering growth factors for regenerative medicine applications. Acta Biomater..

[34] Dereka X.E., Markopoulou C.E., Vrotsos I.A. (2006). Role of growth factors on periodontal repair. Growth Factors.

[35] Miron R.J., Zhang Y. (2018). Autologous liquid platelet rich fibrin: A novel drug delivery system. Acta Biomater..

[36] Green R.J., Usui M.L., Hart C.E., Ammons W.F., Narayanan A.S. (1997). Immunolocalization of platelet-derived growth factor A and B chains and PDGF-alpha and beta receptors in human gingival wounds. J. Periodontal Res..

[37] Mumford J.H., Carnes D.L., Cochran D.L., Oates T.W. (2001). The effects of platelet-derived growth factor-BB on periodontal cells in an in vitro wound model. J. Periodontol..

[38] Boyan L.A., Bhargava G., Nishimura F., Orman R., Price R., Terranova V.P. (1994). Mitogenic and chemotactic responses of human periodontal ligament cells to the different isoforms of platelet-derived growth factor. J. Dent. Res..

[39] Marconi G.D., Fonticoli L., Rajan T.S., Lanuti P., Della Rocca Y., Pierdomenico S.D., Trubiani O., Pizzicannella J., Diomede F. (2021). Transforming Growth Factor-Beta1 and Human Gingival Fibroblast-to-Myofibroblast Differentiation: Molecular and Morphological Modifications. Front. Physiol..

[40] Lichtman M.K., Otero-Vinas M., Falanga V. (2016). Transforming growth factor beta (TGF-β) isoforms in wound healing and fibrosis. Wound Repair Regen..

[41] Schrementi M.E., Ferreira A.M., Zender C., DiPietro L.A. (2008). Site-specific production of TGF-beta in oral mucosal and cutaneous wounds. Wound Repair Regen..

[42] Bao P., Kodra A., Tomic-Canic M., Golinko M.S., Ehrlich H.P., Brem H. (2009). The role of vascular endothelial growth factor in wound healing. J. Surg. Res..

[43] Sadeghi S., Kalhor H., Panahi M., Abolhasani H., Rahimi B., Kalhor R., Mehrabi A., Vahdatinia M., Rahimi H. (2021). Keratinocyte growth factor in focus: A comprehensive review from structural and functional aspects to therapeutic applications of palifermin. Int. J. Biol. Macromol..

[44] Vasita R., Katti D.S. (2006). Growth factor-delivery systems for tissue engineering: A materials perspective. Expert Rev. Med. Devices.

[45] Wang Z., Wang Z., Lu W.W., Zhen W., Yang D., Peng S. (2017). Novel biomaterial strategies for controlled growth factor delivery for biomedical applications. NPG Asia Mater..

[46] Lee K., Silva E.A., Mooney D.J. (2011). Growth factor delivery-based tissue engineering: General approaches and a review of recent developments. J. R. Soc. Interface.

[47] Chen F.M., Zhang M., Wu Z.F. (2010). Toward delivery of multiple growth factors in tissue engineering. Biomaterials.

[48] Burdick J.A., Prestwich G.D. (2011). Hyaluronic acid hydrogels for biomedical applications. Adv. Mater..

[49] Vijayan A., A S., Kumar G.S.V. (2019). PEG grafted chitosan scaffold for dual growth factor delivery for enhanced wound healing. Sci. Rep..

[50] Williams D.F. (2006). To engineer is to create: The link between engineering and regeneration. Trends Biotechnol..

[51] Chen F.M., Jin Y. (2010). Periodontal tissue engineering and regeneration: Current approaches and expanding opportunities. Tissue Eng. Part B Rev..

[52] Kobsa S., Saltzman W.M. (2008). Bioengineering approaches to controlled protein delivery. Pediatr. Res..

[53] Bartold M., Ivanovski S. (2025). Biological processes and factors involved in soft and hard tissue healing. Periodontology 2000.

[54] Rodrigues M., Kosaric N., Bonham C.A., Gurtner G.C. (2019). Wound healing: A cellular perspective. Physiol. Rev..

[55] Golebiewska E.M., Poole A.W. (2015). Platelet secretion: From haemostasis to wound healing and beyond. Blood Rev..

[56] Gillitzer R., Goebeler M. (2001). Chemokines in cutaneous wound healing. J. Leukoc. Biol..

[57] Park J.E., Barbul A. (2004). Understanding the role of immune regulation in wound healing. Am. J. Surg..

[58] Yanez D.A., Lacher R.K., Vidyarthi A., Colegio O.R. (2017). The role of macrophages in skin homeostasis. Pflug. Arch..

[59] Krzyszczyk P., Schloss R., Palmer A., Berthiaume F. (2018). The role of macrophages in acute and chronic wound healing and interventions to promote pro-wound healing phenotypes. Front. Physiol..

[60] Tonnesen M.G., Feng X., Clark R.A. (2000). Angiogenesis in wound healing. J. Investig. Dermatol. Symp. Proc..

[61] Gerhardt H., Golding M., Fruttiger M., Ruhrberg C., Lundkvist A., Abramsson A., Jeltsch M., Mitchell C., Alitalo K., Shima D. (2003). VEGF guides angiogenic sprouting utilizing endothelial tip cell filopodia. J. Cell Biol..

[62] Serini G., Bochaton-Piallat M.L., Ropraz P., Geinoz A., Borsi L., Zardi L., Gabbiani G. (1998). The fibronectin domain ED-A is crucial for myofibroblastic phenotype induction by transforming growth factor-β1. J. Cell Biol..

[63] Bergmeier V., Etich J., Pitzler L., Frie C., Koch M., Fischer M., Rappl G., Abken H., Tomasek J.J., Brachvogel B. (2018). Identification of a myofibroblast-specific expression signature in skin wounds. Matrix Biol..

[64] Tomasek J.J., Gabbiani G., Hinz B., Chaponnier C., Brown R.A. (2002). Myofibroblasts and mechano-regulation of connective tissue remodelling. Nat. Rev. Mol. Cell Biol..

[65] Larjava H., Koivisto L., Häkkinen L., Heino J. (2011). Epithelial integrins with special reference to oral epithelia. J. Dent. Res..

[66] Caley M.P., Martins V.L.C., O’Toole E.A. (2015). Metalloproteinases and wound healing. Adv. Wound Care.

[67] Sorsa T., Tjäderhane L., Konttinen Y.T., Lauhio A., Salo T., Lee H.M., Golub L.M., Brown D.L., Mäntylä P. (2006). Matrix metalloproteinases: Contribution to pathogenesis, diagnosis and treatment of periodontal inflammation. Ann. Med..

[68] Aellos F., Clavelier A., Liu B., Wan A., Salvi G.E., Romandini M., Helms J.A. (2026). Soft-tissue integration of dental implants: Formation, maintenance, and relevance for peri-implant health. J. Periodontal Res..

[69] Häkkinen L., Larjava H., Fournier B.P.J. (2014). Distinct phenotype and therapeutic potential of gingival fibroblasts. Cytotherapy.

[70] Uchimura K., Morimoto-Tomita M., Bistrup A., Lum D.H., Egeblad M., Boudreau N., Werb Z., Rosen S.D. (2006). HSulf-2, an extracellular endoglucosamine-6-sulfatase, selectively mobilizes heparin-bound growth factors and chemokines: Effects on VEGF, FGF-1, and SDF-1. BMC Biochem..

[71] Hinz B. (2015). The extracellular matrix and transforming growth factor-β1: Tale of a strained relationship. Matrix Biol..

[72] Chambrone L., Tatakis D.N. (2015). Periodontal soft tissue root coverage procedures: A systematic review from the AAP Regeneration Workshop. J. Periodontol..

[73] Zucchelli G., Mounssif I. (2015). Periodontal plastic surgery. Periodontology 2000.

[74] Aroca S., Molnár B., Windisch P., Gera I., Salvi G.E., Nikolidakis D., Sculean A. (2013). Treatment of multiple adjacent Miller class I and II gingival recessions with a modified coronally advanced tunnel technique and a collagen matrix or palatal connective tissue graft: A randomized, controlled clinical trial. J. Clin. Periodontol..

[75] Ghanaati S., Schlee M., Webber M.J., Willershausen I., Barbeck M., Balic E., Görlach C., Stupp S.I., Sader R.A., Kirkpatrick C.J. (2011). Evaluation of the tissue reaction to a new bilayered collagen matrix in vivo and its translation to the clinic. Biomed. Mater..

[76] Delgado L.M., Bayon Y., Pandit A., Zeugolis D.I. (2015). To cross-link or not to cross-link? Cross-linking associated foreign body response of collagen-based devices. Tissue Eng. Part B Rev..

[77] Zhang M., Wang M., Zhang C. (2022). Efficacy and safety of acellular dermal matrix versus connective tissue graft for root coverage of Miller’s Class I and II gingival recession: A systematic review and meta-analysis. Ann. Palliat. Med..

[78] Faraj K.A., Brouwer K.M., Geutjes P.J., Versteeg E.M., Wismans R.G., Deprest J.A., Chajra H., Tiemessen D.M., Feitz W.F.J., Oosterwijk E. (2011). The effect of ethylene oxide sterilisation, beta irradiation and gamma irradiation on collagen fibril-based scaffolds. Tissue Eng. Regen. Med..

[79] Matuska A.M., McFetridge P.S. (2015). The effect of terminal sterilization on structural and biophysical properties of a decellularized collagen-based scaffold; implications for stem cell adhesion. J. Biomed. Mater. Res. B Appl. Biomater..

[80] Makadia H.K., Siegel S.J. (2011). Poly lactic-co-glycolic acid (PLGA) as biodegradable controlled drug delivery carrier. Polymers.

[81] Bottino M.C., Thomas V., Schmidt G., Vohra Y.K., Chu T.M., Kowolik M.J., Janowski G.M. (2012). Recent advances in the development of GTR/GBR membranes for periodontal regeneration—A materials perspective. Dent. Mater..

[82] Jin S., Yu Y., Zhang T., Xie D., Zheng Y., Wang C., Liu Y., Xia D. (2024). Surface modification strategies to reinforce the soft tissue seal at transmucosal region of dental implants. Bioact. Mater..

[83] Suesca E., Dias A.M.A., Braga M.E.M., de Sousa H.C., Fontanilla M.R. (2017). Multifactor analysis on the effect of collagen concentration, cross-linking and fiber/pore orientation on chemical, microstructural, mechanical and biological properties of collagen type I scaffolds. Mater. Sci. Eng. C Mater. Biol. Appl..

[84] Tommasato G., Del Fabbro M., Oliva N., Khijmatgar S., Grusovin M.G., Sculean A., Canullo L. (2024). Autogenous graft versus collagen matrices for peri-implant soft tissue augmentation. A systematic review and network meta-analysis. Clin. Oral Investig..

[85] Dadlani S., Joseph B., Anil S. (2024). Efficacy of xenogeneic collagen matrices in augmenting peri-implant soft tissue: A systematic review and meta-analysis. Clin. Exp. Dent. Res..

[86] Cairo F., Rupe C., Cavalcanti R., Landi L., Rupe A., Sforza N.M., Castelluzzo W., Di Martino M., Barbato L. (2026). Cross-linked volume-stable collagen matrix versus connective tissue graft for soft tissue augmentation at implant site: A non-inferiority, multicenter randomized clinical trial. Clin. Oral Implants Res..

[87] Wu F., Jin T. (2008). Polymer-based sustained-release dosage forms for protein drugs, challenges, and recent advances. AAPS PharmSciTech.

[88] Blatt S., Burkhardt V., Kämmerer P.W., Pabst A.M., Sagheb K., Heller M., Al-Nawas B., Schiegnitz E. (2020). Biofunctionalization of porcine-derived collagen matrices with platelet rich fibrin: Influence on angiogenesis in vitro and in vivo. Clin. Oral Investig..

[89] Schröger S.V., Becker J., Kaya S., Rahimi-Nedjat R., Sagheb K., Blatt S. (2026). Boosting angiogenesis experimentally in ovo by biofunctionalizing collagen membranes with platelet-rich fibrin and hyaluronic acid. Int. J. Implant Dent..

[90] Wijayanti D.A., Wirajaya G.N.K.A., Pratiwi N.H., Karina V.M., Murdiastuti K. (2026). Combination of collagen-chitosan hydrogel and injectable platelet-rich fibrin as a biomaterial for bone regeneration. Eur. J. Dent..

[91] Moharamzadeh K., Colley H., Murdoch C., Hearnden V., Chai W.L., Brook I.M., Thornhill M.H., MacNeil S. (2012). Tissue-engineered oral mucosa. J. Dent. Res..

[92] Laschke M.W., Menger M.D. (2016). Prevascularization in tissue engineering: Current concepts and future directions. Biotechnol. Adv..

[93] Williams D.J., Thomas R.J., Hourd P.C., Chandra A., Ratcliffe E., Liu Y., Rayment E.A., Archer J.R. (2012). Precision manufacturing for clinical-quality regenerative medicines. Philos. Trans. R. Soc. A Math. Phys. Eng. Sci..

[94] Sakiyama-Elbert S.E. (2014). Incorporation of heparin into biomaterials. Acta Biomater..

[95] Hettiaratchi M.H., Guldberg R.E., McDevitt T.C. (2016). Biomaterial strategies for controlling stem cell fate via morphogen sequestration. J. Mater. Chem. B.

[96] Marjanovic J., Jurczuk V., Tose L.V., Cintron Diaz Y., Fernandez Lima F., Abdo Abujamra B., Danker S., Jabori S., Singh D., Burgess J.L. (2025). Scaffolds with spatiotemporally controlled growth factor delivery and cyclodextrin-enabled antagonism of growth factor receptor sequestration promote cutaneous wound healing. npj Regen. Med..

[97] Jeon O., Kang S.W., Lim H.W., Chung J.H., Kim B.S. (2006). Long-term and zero-order release of basic fibroblast growth factor from heparin-conjugated poly(L-lactide-co-glycolide) nanospheres and fibrin gel. Biomaterials.

[98] Hersel U., Dahmen C., Kessler H. (2003). RGD modified polymers: Biomaterials for stimulated cell adhesion and beyond. Biomaterials.

[99] Massia S.P., Hubbell J.A. (1991). An RGD spacing of 440 nm is sufficient for integrin alpha V beta 3-mediated fibroblast spreading and 140 nm for focal contact and stress fiber formation. J. Cell Biol..

[100] Massia S.P., Hubbell J.A. (1990). Covalent surface immobilization of Arg-Gly-Asp- and Tyr-Ile-Gly-Ser-Arg-containing peptides to obtain well-defined cell-adhesive substrates. Anal. Biochem..

[101] Palaiologou A.A., Yukna R.A., Moses R., Lallier T.E. (2001). Gingival, dermal, and periodontal ligament fibroblasts express different extracellular matrix receptors. J. Periodontol..

[102] Vallecillo C., Toledano-Osorio M., Vallecillo-Rivas M., Toledano M., Rodriguez-Archilla A., Osorio R. (2021). Collagen matrix vs. autogenous connective tissue graft for soft tissue augmentation: A systematic review and meta-analysis. Polymers.

[103] Ashurko I., Tarasenko S., Magdalyanova M., Bokareva S., Balyasin M., Alimova A., Molchanov N., Zhornik M., Unkovskiy A. (2023). Comparative analysis of xenogeneic collagen matrix and autogenous subepithelial connective tissue graft to increase soft tissue volume around dental implants: A systematic review and meta-analysis. BMC Oral Health.

[104] Cairo F., Barbato L., Tonelli P., Batalocco G., Pagavino G., Nieri M. (2017). Xenogeneic collagen matrix versus connective tissue graft for buccal soft tissue augmentation at implant site. A randomized, controlled clinical trial. J. Clin. Periodontol..

[105] Ashurko I., Tarasenko S., Esayan A., Kurkov A., Mikaelyan K., Balyasin M., Galyas A., Kustova J., Taschieri S., Corbella S. (2022). Connective tissue graft versus xenogeneic collagen matrix for soft tissue augmentation at implant sites: A randomized-controlled clinical trial. Clin. Oral Investig..

[106] Ríos-Osorio N., Orozco-Agudelo M., Cespedes-Moreno I., Contreras-Ibarra M., Guerrero-Torres M., Castro-Calderón A., Grajales M., Jiménez-Peña O. (2026). Comparative outcomes of xenogeneic collagen matrices and autogenous graft-based techniques to increase mucosal thickness and keratinised mucosa width at implant sites: Are autologous grafts still the gold standard? A systematic review and meta-analysis of randomised clinical trials. J. Periodontal Implant Sci..

[107] Galve-Huertas A., Decadt L., García-González S., Hernández-Alfaro F., Aboul-Hosn Centenero S. (2024). Immediate Implant Placement with Soft Tissue Augmentation Using Acellular Dermal Matrix Versus Connective Tissue Graft: A Systematic Review and Meta-Analysis. Materials.

[108] Miron R.J., Fujioka-Kobayashi M., Bishara M., Zhang Y., Hernandez M., Choukroun J. (2017). Platelet-rich fibrin and soft tissue wound healing: A systematic review. Tissue Eng. Part B Rev..

[109] Mancini L., Tarallo F., Quinzi V., Fratini A., Mummolo S., Marchetti E. (2021). Platelet-Rich Fibrin in Single and Multiple Coronally Advanced Flap for Type 1 Recession: An Updated Systematic Review and Meta-Analysis. Medicina.

[110] Rodas M.A.R., Paula B.L., Pazmiño V.F.C., Lot Vieira F.F.D.S., Junior J.F.S., Silveira E.M.V. (2020). Platelet-Rich Fibrin in Coverage of Gingival Recession: A Systematic Review and Meta-Analysis. Eur. J. Dent..

[111] Yavuz A., Güngörmek H.S., Kuru L., Doğan B. (2024). Treatment of multiple adjacent gingival recessions using leucocyte- and platelet-rich fibrin with coronally advanced flap: A 12-month split-mouth controlled randomized clinical trial. Clin. Oral Investig..

[112] Carrera T.M.I., Machado L.M., Soares M.T.R., Passos G.P., Oliveira G.J.P., Ribeiro Júnior N.V., Soares P.B.F., Pigossi S.C. (2023). Root coverage with platelet-rich fibrin or connective tissue graft: A split-mouth randomized trial. Braz. Oral Res..

[113] Silva M.P., Colognese M.R.V., Nassar C.A., Camilotti V. (2026). Comparative analysis of connective tissue graft versus ozone-enriched platelet-rich fibrin in the treatment of Cairo class I gingival recessions: A randomized, double-blind, controlled clinical trial. J. Appl. Oral Sci..

[114] Kaigler D., Cirelli J.A., Giannobile W.V. (2006). Growth factor delivery for oral and periodontal tissue engineering. Expert Opin. Drug Deliv..

[115] Barone S., Bennardo F., Salviati M., Antonelli A., Giudice A. (2025). Evaluation of the usefulness of platelet-rich fibrin (PRF) in mandibular third molar surgery with 3D facial swelling analysis: A split-mouth randomized clinical trial. Head Face Med..

[116] Masuki H., Okudera T., Watanebe T., Suzuki M., Nishiyama K., Okudera H., Nakata K., Uematsu K., Su C.Y., Kawase T. (2016). Growth factor and pro-inflammatory cytokine contents in platelet-rich plasma (PRP), plasma rich in growth factors (PRGF), advanced platelet-rich fibrin (A-PRF), and concentrated growth factors (CGF). Int. J. Implant Dent..

[117] Fujioka-Kobayashi M., Schaller B., Mourão C.F.A.B., Zhang Y., Sculean A., Miron R.J. (2021). Biological characterization of an injectable platelet-rich fibrin mixture consisting of autologous albumin gel and liquid platelet-rich fibrin (Alb-PRF). Platelets.

[118] Kargarpour Z., Nasirzade J., Panahipour L., Miron R.J., Gruber R. (2020). Liquid platelet-rich fibrin and heat-coagulated albumin gel: Bioassays for TGF-β activity. Materials.

[119] Dohan Ehrenfest D.M., Pinto N.R., Pereda A., Jiménez P., Del Corso M., Kang B.S., Nally M., Lanata N., Wang H.L., Quirynen M. (2018). The impact of the centrifuge characteristics and centrifugation protocols on the cells, growth factors, and fibrin architecture of a leukocyte- and platelet-rich fibrin (L-PRF) clot and membrane. Platelets.

[120] Miron R.J., Chai J., Zhang P., Li Y., Wang Y., Mourão C.F.A.B., Sculean A., Fujioka Kobayashi M., Zhang Y. (2020). A novel method for harvesting concentrated platelet-rich fibrin (C-PRF) with a 10-fold increase in platelet and leukocyte yields. Clin. Oral Investig..

[121] Tavelli L., Barootchi S., Rodriguez M.V., Mancini L., Majzoub J., Travan S., Sugai J., Chan H.L., Kripfgans O., Wang H.L. (2022). Recombinant human platelet-derived growth factor improves root coverage of a collagen matrix for multiple adjacent gingival recessions: A triple-blinded, randomized, placebo-controlled trial. J. Clin. Periodontol..

[122] Tavelli L., Ravidà A., Barootchi S., Chambrone L., Giannobile W.V. (2021). Recombinant human platelet-derived growth factor: A systematic review of clinical findings in oral regenerative procedures. JDR Clin. Transl. Res..

[123] Tavelli L., Barootchi S., Rodriguez M.V., Mancini L., Sabri H., Nguyen T., Majzoub J., Travan S., Giannobile W.V. (2025). Stability of root coverage outcomes after soft-tissue augmentation with a collagen matrix with or without rhPDGF-BB: A 3-year triple-blinded, randomised, placebo-controlled trial. J. Clin. Periodontol..

[124] Carney C.M., Rossmann J.A., Kerns D.G., Cipher D.J., Rees T.D., Solomon E.S., Rivera-Hidalgo F., Beach M.M. (2012). A comparative study of root defect coverage using an acellular dermal matrix with and without a recombinant human platelet-derived growth factor. J. Periodontol..

[125] Santamaria M.P., Rossato A., Miguel M.M.V., Bonafé A.C.F., Mathias-Santamaria I.F., Nunes M.P. (2026). Treatment of single gingival recessions using iPRF-biofunctionalized collagen matrix: A randomized clinical trial. J. Periodontol..

[126] Magrin G.L., Michels R., Gil A.C.K., Maier K.B., Mueller B., Gruber R., Benfatti C.A.M. (2026). Volume-stable collagen matrix with or without platelet-rich fibrin impregnation for multiple gingival recessions: A randomized clinical trial. J. Periodontal Res..

[127] Çetiner D., Gökalp Kalabay P., Özdemir B., Çankaya Z.T. (2018). Efficiency of platelet-rich plasma on acellular dermal matrix application with coronally advanced flap in the treatment of multiple adjacent gingival recessions: A randomized controlled clinical trial. J. Dent. Sci..

[128] Chandra R.V., Sneha K., Pushpalatha S., Chakravarthy Y. (2020). Efficacy of recombinant human fibroblast growth factor 2 impregnated absorbable collagen membrane in the treatment of Miller’s Class I and II gingival recession defects Preliminary results from the first in human clinical trial. J. Indian Soc. Periodontol..

[129] Singh K., Sharma S., Singh K.S., Varshney A., Agarwal K., Sahai T. (2026). Management of Miller’s Class I and II Gingival Recession with FGF-2 Impregnated versus Plain Bioresorbable Collagen Membrane: A Randomised Controlled Trial. J. Clin. Diagn. Res..

[130] Sankar D., Arumuganainar D., Baskaran S., Gopalakrishnan S. (2026). Tissue-engineered approach using autologous fibroblast-seeded volume-stable collagen matrix for managing gingival recession—A randomized controlled clinical trial. J. Oral Biol. Craniofac Res..

[131] Cardaropoli D., Roffredo A., Tamagnone L., Gaveglio L., Araujo M. (2025). Polynucleotides and Hyaluronic Acid Mixture with Volume Stable Collagen Matrix for Coverage of Multiple Gingival Recession: A Case Series. Int. J. Periodontics Restor. Dent..

[132] Discepoli N., Mirra R., Ferrari M. (2019). Efficacy of Enamel Derivatives to Improve Keratinized Tissue as Adjunct to Coverage of Gingival Recessions: A Systematic Review and Meta-Analysis. Materials.

[133] Dias A.T., de Menezes C.C., Kahn S., Fischer R.G., da Silva Figueredo C.M., Fernandes G.V.O. (2022). Gingival recession treatment with enamel matrix derivative associated with coronally advanced flap and subepithelial connective tissue graft: A split-mouth randomized controlled clinical trial with molecular evaluation. Clin. Oral Investig..

[134] Rasperini G., Roccuzzo M., Francetti L., Acunzo R., Consonni D., Silvestri M. (2011). Subepithelial connective tissue graft for treatment of gingival recessions with and without enamel matrix derivative: A multicenter, randomized controlled clinical trial. Int. J. Periodontics Restor. Dent..

[135] Stähli A., Duong H.Y., Imber J.C., Roccuzzo A., Salvi G.E., Katsaros C., Ramseier C.A., Sculean A. (2022). Recession coverage using the modified coronally advanced tunnel and connective tissue graft with or without enamel matrix derivative: 5-year results of a randomised clinical trial. Clin. Oral Investig..

[136] Sangiorgio J.P.M., Neves F.L.d.S., Rocha dos Santos M., França-Grohmann I.L., Casarin R.C.V., Casati M.Z., Santamaria M.P., Sallum E.A. (2017). Xenogenous Collagen Matrix and/or Enamel Matrix Derivative for Treatment of Localized Gingival Recessions: A Randomized Clinical Trial. Part I: Clinical Outcomes. J. Periodontol..

[137] Shaikh M.S., Lone M.A., Matabdin H., Lone M.A., Soomro A.H., Zafar M.S. (2021). Regenerative potential of enamel matrix protein derivative and acellular dermal matrix for gingival recession: A systematic review and meta-analysis. Proteomes.

[138] Rieder M., Wimmer G., Sokolowski A., Sokolowski A., Payer M., Arefnia B. (2024). Treatment of localized gingival recession with an enamel matrix protein-coated xenogeneic dermal matrix: A randomized controlled trial. Materials.

[139] Schmitt C.M., Schlegel K.A., Gammel L., Moest T. (2021). Volumetric soft tissue alterations in the early healing phase after peri-implant soft tissue contour augmentation with a porcine collagen matrix versus the autologous connective tissue graft: A controlled clinical trial. J. Clin. Periodontol..

[140] Zeltner M., Jung R.E., Hämmerle C.H.F., Hüsler J., Thoma D.S. (2017). Randomized controlled clinical study comparing a volume-stable collagen matrix to autogenous connective tissue grafts for soft tissue augmentation at implant sites: Linear volumetric soft tissue changes up to 3 months. J. Clin. Periodontol..

[141] Guiha R., el Khodeiry S., Mota L., Caffesse R. (2001). Histological evaluation of healing and revascularization of the subepithelial connective tissue graft. J. Periodontol..

[142] Hujoel P.P., DeRouen T.A. (1992). Validity issues in split-mouth trials. J. Clin. Periodontol..

[143] Lesaffre E., Pihlstrom B., Needleman I., Worthington H. (2009). The design and analysis of split-mouth studies: What statisticians and clinicians should know. Stat. Med..

[144] Tramèr M.R., Reynolds D.J., Moore R.A., McQuay H.J. (1997). Impact of covert duplicate publication on meta-analysis: A case study. Br. Med. J..

[145] Sbricoli L., Guazzo R., Annunziata M., Gobbato L., Bressan E., Nastri L. (2020). Selection of collagen membranes for bone regeneration: A literature review. Materials.

[146] Vallecillo C., Toledano-Osorio M., Vallecillo-Rivas M., Toledano M., Osorio R. (2021). In vitro biodegradation pattern of collagen matrices for soft tissue augmentation. Polymers.

[147] Koteneva P., Kosheleva N., Fayzullin A., Khristidis Y., Bunin E., Efremov Y., Zhukova D., Tkachev S., Kulova A., Rasulov T. (2025). From bench to chairside: Collagen scaffolds in combination with mesenchymal stromal cells for gingival augmentation. Dent. J..

[148] Delgado L.M., Pandit A., Zeugolis D.I. (2014). Influence of sterilisation methods on collagen-based devices stability and properties. Expert Rev. Med. Devices.

[149] Bracey D.N., Seyler T.M., Jinnah A.H., Lively M.O., Willey J.S., Smith T.L., Van Dyke M.E., Whitlock P.W. (2018). A Decellularized Porcine Xenograft-Derived Bone Scaffold for Clinical Use as a Bone Graft Substitute: A Critical Evaluation of Processing and Structure. J. Funct. Biomater..

[150] Sant S., Hancock M.J., Donnelly J.P., Iyer D., Khademhosseini A. (2010). Biomimetic gradient hydrogels for tissue engineering. Can. J. Chem. Eng..

[151] Fujioka-Kobayashi M., Miron R.J., Miron R.J., Choukroun J. (2017). Biological Components of Platelet Rich Fibrin: Growth Factor Release and Cellular Activity. Platelet Rich Fibrin in Regenerative Dentistry: Biological Background and Clinical Indications.

[152] Ramamoorth M., Narvekar A. (2015). Non viral vectors in gene therapy—An overview. J. Clin. Diagn. Res..

[153] Jing L., Wang H.Y., Zhang N., Zhang W.J., Chen Y., Deng D.K., Li X., Chen F.M., He X.T. (2025). Critical roles of extracellular vesicles in periodontal disease and regeneration. Stem Cells Transl. Med..

[154] Wei M., Gao Y., Li X., Serpe M.J. (2017). Stimuli-responsive polymers and their applications. Polym. Chem..

[155] Li W., Li M., Cai T., Ding Y., Tian W., Guo S. (2025). Intelligent biomaterials for periodontitis and peri-implantitis therapies: Stimuli-responsive strategies targeting inflammation and regeneration. J. Mater. Chem. B.

[156] Pereira M.M.A., Piazza R.D., Barbugli P.A., Tagit O., van den Beucken J.J.J.P., Das A., Biffe C.A., Barão V.A.R., da Cruz S.B.A., Ervolino E. (2026). Targeted infection control and tissue integration via pH-sensitive smart coatings on implant surfaces. ACS Appl. Mater. Interfaces.

[157] Verza B.S., van den Beucken J.J.J.P., Brandt J.V., Jafelicci Junior M., Barão V.A.R., Piazza R.D., Tagit O., Spolidorio D.M.P., Vergani C.E., de Avila E.D. (2021). A long-term controlled drug-delivery with anionic beta cyclodextrin complex in layer-by-layer coating for percutaneous implants devices. Carbohydr. Polym..

[158] Saravanakumar G., Kim J., Kim W.J. (2017). Reactive-oxygen-species-responsive drug delivery systems: Promises and challenges. Adv. Sci..

[159] Shafiq M., Chen Y., Hashim R., He C., Mo X., Zhou X. (2021). Reactive Oxygen Species-Based Biomaterials for Regenerative Medicine and Tissue Engineering Applications. Front. Bioeng. Biotechnol..

[160] Sczepanik F.S.C., Grossi M.L., Casati M., Goldberg M., Glogauer M., Fine N., Tenenbaum H.C. (2020). Periodontitis is an inflammatory disease of oxidative stress: We should treat it that way. Periodontology 2000.

[161] Bostanci N., Belibasakis G.N. (2012). *Porphyromonas gingivalis*: An invasive and evasive opportunistic oral pathogen. FEMS Microbiol. Lett..

[162] Thambi T., Park J.H., Lee D.S. (2016). Hypoxia-responsive nanocarriers for cancer imaging and therapy: Recent approaches and future perspectives. Chem. Commun..

[163] Humphrey J.D., Dufresne E.R., Schwartz M.A. (2014). Mechanotransduction and extracellular matrix homeostasis. Nat. Rev. Mol. Cell Biol..

[164] Kapat K., Shubhra Q.T.H., Zhou M., Leeuwenburgh S. (2020). Piezoelectric nano-biomaterials for biomedicine and tissue regeneration. Adv. Funct. Mater..

[165] Majumder J., Minko T. (2021). Multifunctional and stimuli-responsive nanocarriers for targeted therapeutic delivery. Expert Opin. Drug Deliv..

[166] Wan Z., Zhang P., Liu Y., Lv L., Zhou Y. (2020). Four-dimensional bioprinting: Current developments and applications in bone tissue engineering. Acta Biomater..

[167] Discher D.E., Janmey P., Wang Y.L. (2005). Tissue cells feel and respond to the stiffness of their substrate. Science.

[168] Smith P.C., Cáceres M., Martínez C., Oyarzún A., Martínez J. (2015). Gingival wound healing: An essential response disturbed by aging?. J. Dent. Res..

[169] Köseoğlu S., Duran İ., Sağlam M., Bozkurt S.B., Kırtıloğlu O.S., Hakkı S.S. (2013). Efficacy of collagen membrane seeded with autologous gingival fibroblasts in gingival recession treatment: A randomized, controlled pilot study. J. Periodontol..

[170] Milinkovic I., Aleksic Z., Jankovic S., Popovic O., Bajic M., Cakic S., Lekovic V. (2015). Clinical application of autologous fibroblast cell culture in gingival recession treatment. J. Periodontal Res..

[171] Sanchez N., Vignoletti F., Sanz-Martin I., Coca A., Nuñez J., Maldonado E., Sanz-Esporrin J., Hernando-Pradíes I., Santamaría S., Herrera D. (2022). Cell therapy based on gingiva-derived mesenchymal stem cells seeded in a xenogeneic collagen matrix for root coverage of RT1 gingival lesions: An in vivo experimental study. Int. J. Mol. Sci..

[172] Bertolin M., Ferrari S., Breda C., Ferrari B., Ponzin D., Barbaro V. (2020). GMP-compliant manufacturing process of an advanced therapy medicinal product based on conjunctival epithelial cells. Res. Sq..

[173] Hourd P., Chandra A., Medcalf N., Williams D.J. (2014). Regulatory challenges for the manufacture and scale-out of autologous cell therapies. StemBook.

[174] Hourd P., Medcalf N., Segal J., Williams D.J. (2015). A 3D bioprinting exemplar of the consequences of the regulatory requirements on customized processes. Regen. Med..

[175] Manglaris C., Karipidou N., Manolakaki E., Koummati E., Choli-Papadopoulou T., Koidis P.T., Aggeli A., Michailidis N. (2026). Ozone as a Next-Generation Sterilization Process in Tissue Engineering and Regenerative Medicine: Critically Bridging Product Stability, Safety and Clinical Translation. Molecules.

[176] Coopman K., Medcalf N. (2008). From production to patient: Challenges and approaches for delivering cell therapies. StemBook.

[177] Nowak-Sliwinska P., Segura T., Iruela-Arispe M.L. (2014). The chicken chorioallantoic membrane model in biology, medicine and bioengineering. Angiogenesis.

[178] Blanco-Elices C., España-Guerrero E., Mateu-Sanz M., Sánchez-Porras D., García-García Ó.D., Sánchez-Quevedo M.D.C., Fernández-Valadés R., Alaminos M., Martín-Piedra M.Á., Garzón I. (2020). In vitro generation of novel functionalized biomaterials for use in oral and dental regenerative medicine applications. Materials.

[179] Sieira Gil R., Pagés C.M., Díez E.G., Llames S., Fuertes A.F., Vilagran J.L. (2015). Tissue-engineered oral mucosa grafts for intraoral lining reconstruction of the maxilla and mandible with a fibula flap. J. Oral Maxillofac. Surg..

[180] Martin-Piedra M.A., Albendin-Moreno M., Olivares-Abril A., Paez-Yepes S.V.J., Rivera-Izquierdo M., Sánchez-Porras D., Fernández-Valadés R., España-López A., Alaminos M., Garzon I. (2026). Tissue engineering for oral mucosa biofabrication: A systematic review and meta-analysis. Tissue Eng. Part B Rev..

